# Pregranulosa cell–derived FGF23 protects oocytes from premature apoptosis during primordial follicle formation by inhibiting p38 MAPK in mice

**DOI:** 10.1016/j.jbc.2023.104776

**Published:** 2023-05-02

**Authors:** Zijian Zhu, Shaogang Qin, Tuo Zhang, Meina He, Wenying Zheng, Ting Zhao, Meng Gao, Ziqi Chen, Bo Zhou, Guoliang Xia, Chao Wang

**Affiliations:** 1 State Key Laboratory of Farm Animal Biotech Breeding, College of Biological Sciences, China Agricultural University, Beijing, China; 2 Guizhou Provincial Key Laboratory of Pathogenesis and Drug Research on Common Chronic Diseases, Department of Physiology, College of Basic Medicine, Guizhou Medical University, Guiyang, Guizhou Province, China; 3 College of Basic Medicine, Guizhou Medical University, Guiyang, Guizhou Province, China; 4 Key Laboratory of Ministry of Education for Conservation and Utilization of Special Biological Resources in the Western China, College of Life Science, Ningxia University, Yinchuan, China

**Keywords:** FGF23, FGFR1, p38 MAPK, apoptosis, primordial follicle formation, oocyte

## Abstract

A large number of oocytes in the perinatal ovary in rodents get lost for unknown reasons. The granulosa cell–oocyte mutual communication is pivotal for directing formation of the primordial follicle; however, little is known if paracrine factors participate in modulating programmed oocyte death perinatally. We report here that pregranulosa cell–derived fibroblast growth factor 23 (FGF23) functioned in preventing oocyte apoptosis in the perinatal mouse ovary. Our results showed that FGF23 was exclusively expressed in pregranulosa cells, while fibroblast growth factor receptors (FGFRs) were specifically expressed in the oocytes in perinatal ovaries. FGFR1 was one of the representative receptors in mediating FGF23 signaling during the formation of the primordial follicle. In cultured ovaries, the number of live oocytes declines significantly, accompanied by the activation of the p38 mitogen-activated protein kinase signaling pathway, under the condition of FGFR1 disruption by specific inhibitors of FGFR1 or silencing of *Fgf23*. As a result, oocyte apoptosis increased and eventually led to a decrease in the number of germ cells in perinatal ovaries following the treatments. In the perinatal mouse ovary, pregranulosa cell–derived FGF23 binds to FGFR1 and activates at least the p38 mitogen-activated protein kinase signaling pathway, thereby regulating the level of apoptosis during primordial follicle formation. This study reemphasizes the importance of granulosa cell–oocyte mutual communication in modulating primordial follicle formation and supporting oocyte survival under physiological conditions.

In female mammals, primordial follicle is the basic structural and functional unit of reproduction, which provides an essential structural basis and microenvironment to maintain a dormant state of oocyte in ovary. The primordial follicle pool is considered to be nonrenewable reproductive resources of female mammals (1, 2). Once the pool is established, the total number of follicles in the ovary reaches to peak (3, 4). More than half of oocytes are lost during the formation of primordial follicles (5, 6). The fate of oocytes in the fetal ovary seems to be determined by different protective strategies through the timely control of apoptosis or autophagy. According to present studies, the oocytes have developed mature protective mechanisms before meiosis prophase I has been successfully achieved, including the progressively controlled activity of glycogen synthase kinase-3 beta (GSK-3β), the levels of lysine-specific demethylase 1, and the balanced relationship between X-linked inhibitor of apoptosis and CASP9, to prevent premature oocyte loss (7, 8). Despite that, the endogenous and exogenous factors that cause the two typical programmed cell death (PCD) in oocytes remain elusive (6, 9).

Hormones and growth factors are vital for blocking default cell death pathway (10). For instances, S100A8 is an oocyte-expressed chemotaxin which functions by directing ovarian somatic cell (OSC) migration during primordial follicle formation (11, 12). Oocyte-derived factor Jagged1 governs the primordial follicle formation by controlling the development of ovarian pregranulosa cells via ADAM10–Notch signaling (13). Besides, *Zp1*, *Zp2*, *Zp3*, *Figa*, *Cx43*, and other genes related to oocyte/follicle function are parts of the determining factors for normal primordial follicle formation (14). Taken together, the recruitment of OSCs essential for establishment of the ovarian reserve depends on multiple paracrine factors derived from oocytes. Although one of our studies have uncovered the role of progesterone in modulating primordial follicle formation (15), little is known if extracellular molecules, especially paracrine molecules, get involved in oocyte death during formation of the primordial follicles.

The generic mitogen-activated protein kinases (MAPKs) have been proved to be pivotal for regulating cellular growth, proliferation, differentiation, migration, and apoptosis (16, 17). This signaling pathway consists of the extracellular signal–related kinases (ERK1/2), Jun amino-terminal kinases (JNK1/2/3), p38 MAPK, and ERK5. Abundant evidences indicated that p38 MAPK involves in apoptosis as well (18, 19, 20, 21). Studies have approved that inhibition of caspases blocks p38 MAPK activation through *Fas* crosslinking (22). Alternatively, overexpression of dominant active mitogen-activated protein kinase kinase 6 also induce caspase activity and cell death, implying that p38 may function both upstream and downstream of caspases in apoptosis (23). Therefore, the role of p38 MAPK in apoptosis could be both cell type and stimulus dependent (24). Whether p38 MAPK plays important roles in the oocytes during primordial follicle formation remains elusive.

Fibroblast growth factor (FGF) plays significant roles not only in the oocyte survival but in granulosa cell differentiation in mammals (25, 26, 27, 28). Of all the 22 members of FGFs, FGF23 was firstly found in patients with autosomal dominant hypophosphatemic rickets (29, 30). As a pleiotropic multiple functional hormone, FGF23 is involved in the phosphate reabsorption, the regulation of vitamin D production, and the conserving of calcium and sodium level in the kidney (31, 32, 33, 34). There are four receptors of FGFs available (35, 36, 37). At physiological conditions, α-Klotho improves the binding affinity of FGF23 to fibroblast growth factor receptors (FGFRs) (38, 39, 40). Interestingly, mice with depleted *Fgf23* display significantly high serum phosphate with increased renal phosphate reabsorption, premature death, kidney and bone defects, and infertility (41, 42). However, how does FGF23 correlates to fertility remains unknown so far.

Here, we have shown that pregranulosa cell–derived FGF23, which binds to FGFR1, regulates the level of apoptosis by activating the p38 MAPK signaling pathway and contributes to determining the fate of oocyte during primordial follicle formation in mice ovary.

## Results

### FGF23 prevents premature oocyte loss during primordial follicle formation

**Figure 1:**
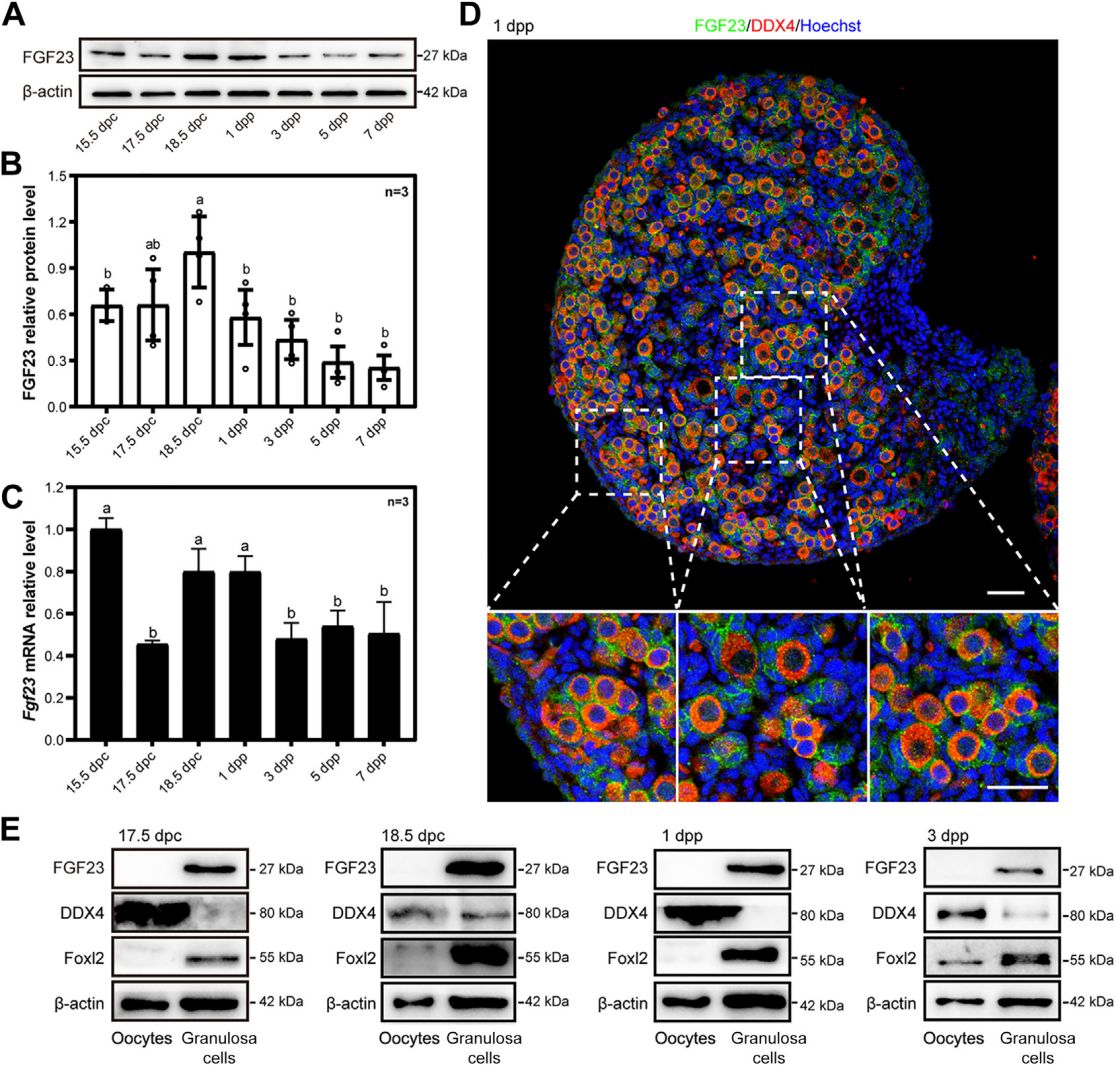
**The expression pattern of FGF23 in mouse ovaries before and after birth.***A* and *B*, the level of FGF23 protein expression in mouse ovaries was measured from 15.5 dpc to seven dpp by Western blotting. The content of FGF23 increased markedly at 18.5 dpc and reduced until seven dpp. n = 3 biologically independent experiments. Data are presented as mean ± SD, and different letters (a, b) indicate significant differences among groups (two-sided ANOVA test), *p* (a, b) < 0.05. *C*, the mRNA expressions of *Fgf23* in mouse ovaries were assessed by qRT-PCR. The expression level of *Fgf23* mRNA was increased at 18.5 dpc. n = 3 biologically independent experiments. Data are presented as mean ± SD, and different letters (a, b) indicate significant differences among groups (two-sided ANOVA test), *p* (a, b) < 0.05. *D*, cellular localization of FGF23 in mouse ovaries. Newborn mouse ovaries were stained for FGF23 (*green*) and the oocyte marker DDX4 (*red*) at one dpp. The nuclei were dyed with a Hoechst counterstain (*blue*). FGF23 was mainly localized to the cytoplasm of granulosa cells in either primordial follicles or growing follicles. The scale bar represents 50 μm. *E*, the protein level of FGF23 in oocytes and granulosa cells. FGF23 was mainly expressed in the granulosa cells. DDX4, DEAD-box helicase 4; FGF, fibroblast growth factor; qRT-PCR, quantitative reverse transcription-PCR.

In order to address the physiological significance of FGF23 during primordial follicle formation, we firstly detected the location and expression pattern of FGF23 in ovaries around the time of birth in mice. The results showed the expression level of FGF23 retains at a relative higher level from 18.5 days postcoitum (dpc) to 1 day postpartum (dpp, Fig. 1, *A* and *B*). The conclusion was reapproved by our quantitative reverse transcription-PCR (qRT-PCR) results (Fig. 1*C*). Immunofluorescence staining results showed that FGF23 was predominantly localized to the cytoplasm of (pre)-granulosa cells in mice ovaries (Fig. 1*D*). Further, to confirm the localization of FGF23 protein within the ovaries, we isolated mouse ovarian granulosa cells and oocytes from the ovaries that have developed to different stages. DEAD-box helicase 4 (DDX4) and Forkhead box L2 (FOXL2) are specifically expressed by oocytes and granulosa cells, respectively. The results showed that the expression level of FGF23 was much higher in the granulosa cells than in the oocytes (Figs. 1*E* and S1).

**Figure 2:**
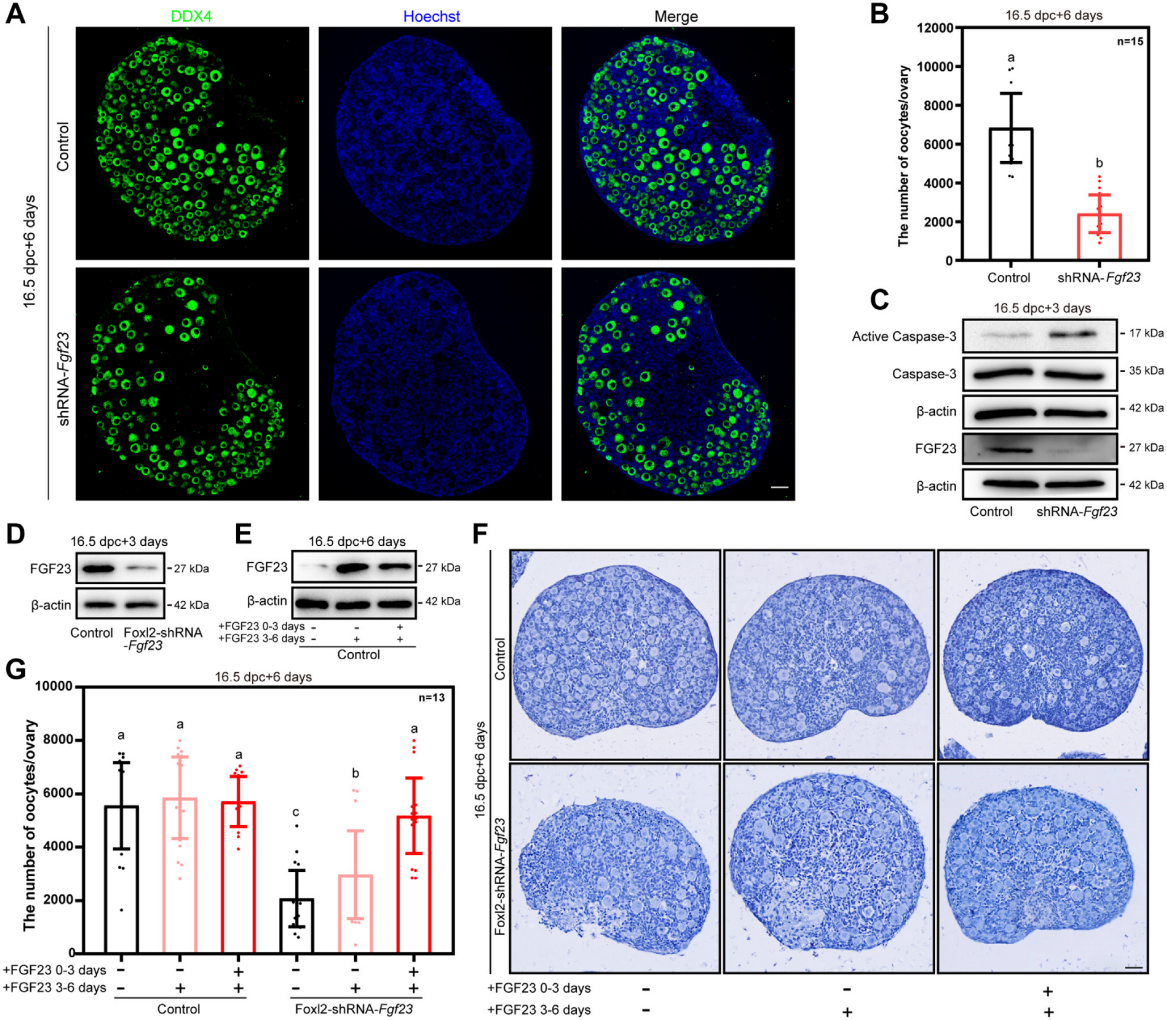
**FGF23 protects oocytes from mass loss during the formation of primordial follicles.***A* and *B*, *Fgf23* knockdown decreased the number of oocytes in mouse ovaries. The mouse ovaries were stained for DDX4 (*green*) and Hoechst (*blue*) after 6 days of culture. The scale bars represent 50 μm (*A*). Quantification of the oocytes per ovary was carried out after 6 days of culture. n = 15. Mice ovaries were taken from at least three biologically independent experiments. Data are presented as mean ± SD, and different letters (a, b) indicate significant differences among groups (two-sided ANOVA test), *p* (a, b) < 0.05 (*B*). *C*, the level of FGF23 protein expression in ovaries was significantly downregulated after *Fgf23* knockdown. *D*, FGF23 protein levels were clearly lower in *Foxl2*-shRNA-*Fgf23* than in the controls after 3 days of culture. *E*, FGF23 protein levels were upregulated after adding FGF23 than in controls after 6 days of culture. *F* and *G*, supplementing FGF23 effectively rescued the oocyte loss caused by *Fgf23* knockdown in the granulosa cells. The scale bars represent 50 μm (*F*). Quantification of oocytes was carried out in control and *Foxl2*-shRNA-*Fgf23*–treated ovaries. n = 13. Mice ovaries were taken from at least three biologically independent experiments. Data are presented as mean ± SD, and different letters (a–c) indicate significant differences among groups (two-sided ANOVA test), *p* (a, b) < 0.05, *p* (a, c) < 0.05, *p* (b, c) < 0.05 (*G*). DDX4, DEAD-box helicase 4; FGF, fibroblast growth factor.

To study the function of FGF23 in regulating primordial follicle formation, we established an *in vitro* culture model of perinatal mouse ovaries pretreated with or without foreign plasmid injection (Fig. S2). To knockdown the *Fgf23* gene, shRNA*-Fgf23* was cloned into pSicoR-GFP (43). Based on this model, we constructed *Fgf23*-shRNA vectors (shRNA-*Fgf23*) and injected the shRNA-*Fgf*23 plasmids into 16.5 dpc ovaries and cultured for 3 days (equal to 1 dpp *in vivo*) (Fig. S3). We then applied the plasmid with the highest knockdown efficiency to perform the subsequent experiments. It showed that the number of available oocytes in the shRNA-*Fgf23* group were significantly less than the control group after RNAi treatment for 6 days (equal to 4 dpp *in vivo*) (Fig. 2, *A* and *B*). Additionally, specific knocking down of *Fgf23* raised the level of cellular apoptosis in the cultured tissues (Fig. 2*C*). Collectively, these results implied that silencing of *Fgf23* accelerated the loss of primordial follicles in perinatal ovaries *in vitro*.

### Pregranulosa cell–derived FGF23 prevents oocyte loss

To further verify if granulosa cell–derived FGF23 contributes to rescue oocyte death, we conducted a granulosa cell–specific knockdown of *Fgf23* (*Foxl2*-shRNA-*Fgf23*). We injected the negative control vector (control) and the *Foxl2*-shRNA-*Fgf23* vector into 16.5 dpc ovaries, respectively. Both the knockdown efficiency of *Fgf23* and the primordial follicle formation of the cultured ovaries were examined, which showed that the FGF23 protein level was significantly decreased after 3 days of culture in the *Foxl2*-shRNA-*Fgf23* group (Fig. 2*D*). The number of the oocytes was significantly less than the control in the 6-days culture group (Fig. 2*G*). Subsequently, the pure FGF23 protein was supplemented into the culture media either immediately or 3 days after injection of *Foxl2*-shRNA-*Fgf23*. The level of FGF23 protein in each group was detected after 6 days. The results showed that the level of the FGF23 protein increased in both culture strategies, namely supplementing FGF23 for either the first 3 days (0–3 days period) or for the last 3 days (3–6 days) (Fig. 2*E*). In addition, the loss of oocytes by RNAi was partially rescued by FGF23 after 3 days of culture, while the loss of oocytes could be effectively rescued by replenishing FGF23 immediately after RNAi (Fig. 2, *F* and *G*). These results further proved that FGF23 does play an important role in regulating primordial follicle formation in mice.

Furthermore, to verify if additional FGF23 contributes to preserve more germ cells, we cultured 16.5 dpc ovaries with various concentrations of FGF23 protein for 6 days before histological analysis. The results showed that the number of total follicles within each ovary in different groups was comparable to the control (Fig. S4), which implies that excessive FGF23 may reach a saturation state with no significant effect on the number of primordia follicles.

### At least one of the FGFRs participates in mediating the function of FGF23 during primordial follicle formation *in vitro*

**Figure 3:**
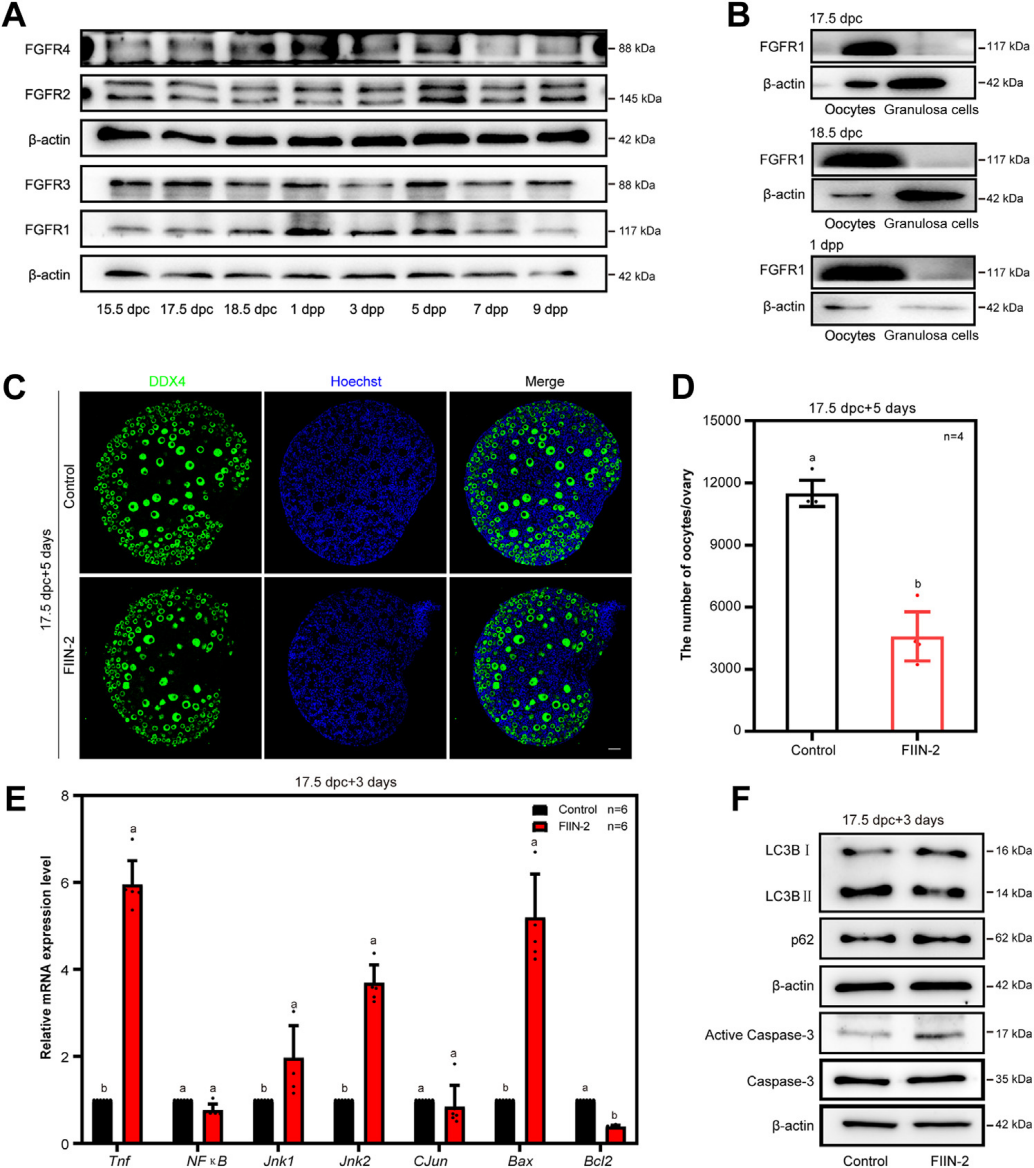
**FGFRs play a regulatory role in the formation of primordial follicles.***A*, the protein levels of FGFR1 through FGFR4 in the mouse ovaries from 15.5 dpc to nine dpp. *B*, localization of FGFR1 in mouse ovaries. FGFR1 was mainly localized to the oocytes. *C* and *D*, inhibition of FGFRs by FIIN-2, one of the nonspecific inhibitors of FGFRs, led to a decrease in the number of germ cells. The mouse ovaries were stained for DDX4 (*green*) and Hoechst (*blue*) after 5 days of culture. The scale bars represent 50 μm (*C*). Quantification of the oocytes per ovary was carried out after 5 days of culture. n = 4 biologically independent experiments. Data are presented as mean ± SD, and different letters (a, b) indicate significant differences among groups (two-sided ANOVA test), *p* (a, b) < 0.05 (*D*). *E*, the mRNA expression levels after inhibition of FGFRs in mouse ovaries were assessed by qRT-PCR. n = 6 biologically independent experiments. Data are presented as mean ± SD, and different letters (a, b) indicate significant differences among groups (two-sided ANOVA test), *p* (a, b) < 0.05. *F*, the apoptosis levels were increased in FIIN-2–treated ovaries as compared to the control. FGFRs, fibroblast growth factor receptors; qRT-PCR, quantitative reverse transcription-PCR.

To investigate the function of FGFRs in primordial follicle formation, we detect the cellular localization and expressing dynamics of FGFRs in ovaries around the time of birth, respectively. The results showed that the expression level of FGFR1 and FGFR4 in the ovaries retain at a relative higher level from 18.5 dpc to 1 dpp, while the expression levels of FGFR2 and FGFR3 did not change significantly from 15.5 dpc to 9 dpp (Figs. 3*A* and S5*A*). The results were further approved by qRT-PCR analysis, which showed the expressions of the mRNA of *Fgfr1* and *Fgfr4* retain at relative higher level from 18.5 dpc to 3 dpp. On the contrary, the expression levels *Fgfr2* and *Fgfr3* decreased before birth but increased after birth (Fig. S5*B*). We next studied whether FGFRs were involved in the process of primordial follicle formation in ovaries. Based on the culture model, 17.5 dpc ovaries were cultured with FIIN-2, one of pan-FGFRs inhibitors, for 5 days (equal to 4 dpp *in vivo*). The statistical analysis of total ovaries showed that inhibition of FGFRs led to a decrease in the number of oocytes (Fig. 3, *C* and *D*). To detect the cause of the loss of oocytes, we cultured 17.5 dpc ovaries for 3 days. Both reverse transcription-PCR as well as Western blotting results showed that apoptosis levels were upregulated in the ovaries after *in vitro* inhibition of FGFRs, whereas, there was no significant change in autophagy levels (Fig. 3, *E* and *F*).

In order to clarify if inhibition of FGFRs would affect oocyte meiosis progression in perinatal mice ovaries, 13.5 dpc ovaries were cultured with FIIN-2 for 4 days (equal to 17.5 dpc *in vivo*). The results showed that the ratio of colocation of Y box Protein 2 (MSY2) and DDX4 between the FIIN-2 group and the control was insignificant (Fig. S6, *A* and *B*), implying that inhibition of the FGFRs in fetal mice ovaries may not influence the progress of oocyte meiosis.

### FGFR1 is the main binding receptor of FGF23, and both of them are involved in the regulation of oocyte number and primordial follicle formation

**Figure 4:**
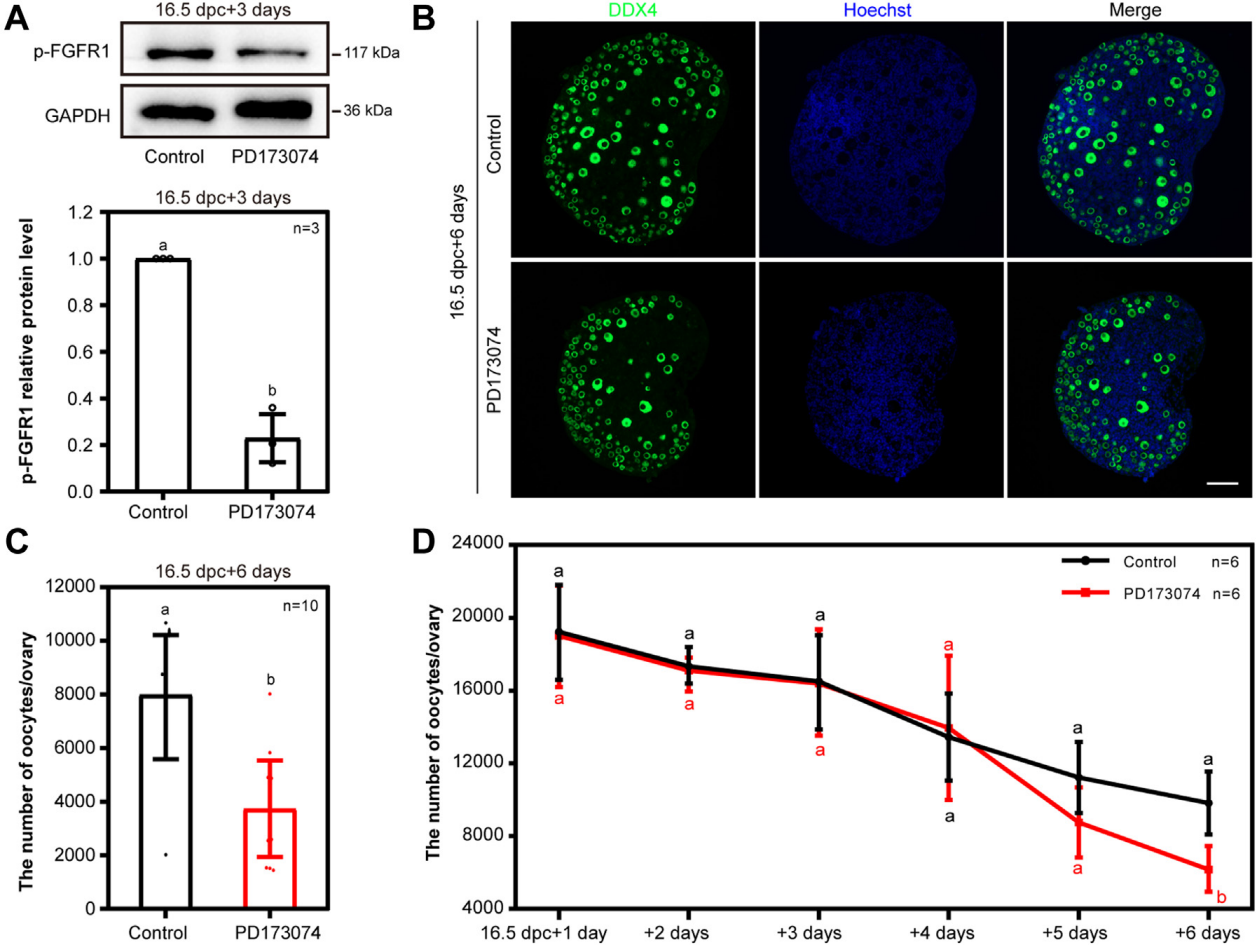
**FGFR1 is essential for the survival of oocytes in the perinatal mice.***A*, the expression level of p-FGFR1 in cultured ovaries was significantly suppressed by PD173074, the specific inhibitor of FGFR1. n = 3 biologically independent experiments. Data are presented as mean ± SD, and different letters (a, b) indicate significant differences among groups (two-sided ANOVA test), *p* (a, b) < 0.05. *B* and *C*, supplementation with PD173074 significantly reduced the oocytes number. The mouse ovaries were stained for DDX4 (*green*) and Hoechst (*blue*) after 6 days of culture. The scale bar represents 50 μm (*B*). Quantification of the oocytes per ovary was carried out after 6 days of culture. n = 10. Mice ovaries were taken from at least three biologically independent experiments. Data are presented as mean ± SD, and different letters (a, b) indicate significant differences among groups (two-sided ANOVA test), *p* (a, b) < 0.05 (*C*). *D*, the time-dependent inhibitory effects of PD173074 on the oocytes number. n = 6. Mice ovaries were taken from at least three biologically independent experiments. Data are presented as mean ± SD, and different letters (a and b) indicate significant differences among groups (two-sided ANOVA test), *p* (a, b) < 0.05. DDX4, DEAD-box helicase 4; FGFR, fibroblast growth factor receptor; p-FGFR1, phosphorylated FGFR1.

In order to identify the most important FGFRs in the regulation of oocyte number during primordial follicle formation, we further specifically inhibited either FGFR1 or FGFR4 *in vitro*. Briefly, 16.5 dpc ovaries were cultured with or without PD173074, one of the specific inhibitors of FGFR1, for 3 days. The results showed that the level of the phosphorylated FGFR1 protein was decreased in PD173074 group as compared to the control (Fig. 4*A*). Moreover, after 6 days of culture by PD173074, much fewer oocytes were available in the experimental group than that in the control ovaries (equal to 4 dpp *in vivo*) (Fig. 4, *B* and *C*). Then, the dynamic change of oocytes number influenced by PD173074 was recorded after 16.5 dpc ovaries were cultured for different days. The results showed that remarkable influence of FGFR1 on oocytes number inhibition showed up on the fourth day of culture (Fig. 4*D*). However, there was no significant change in the number of oocytes if FGFR4 was specifically inhibited by H3B-6527 (Fig. S7). The results showed that FGFR1 is more important than FGFR4 in mediating FGF23 signaling in perinatal mouse ovaries.

Then, we carried out a more detailed detection on the positioning of FGFR1. The results of granulosa cells and oocytes isolated from different developmental stages of mouse ovaries showed that the expression of FGFR1 in the oocytes was significantly higher than that in the granulosa cells (Figs. 3*B* and S5*C*), suggesting that FGF23 is secreted from granulosa cells and acts on oocytes as a paracrine factor to protect oocyte premature loss during primordial follicle formation.

To further verify the function of FGFR1, we performed the *in vitro* reconstitution system. The ovaries were cultured for 4 days with PD173074 and then digested into single cells for reconstruction (Fig. S8*A*). The statistical analysis showed that inhibition of FGFR1 significantly influenced primordial follicle formation (Fig. S8, *B*–*D*). Then, we selected the burosumab (neutralizing antibody of FGF23), FNab10417 (neutralizing antibody of FGFR1), and 6F10 (neutralizing antibody of FGFR1) to further examine the role of FGF23-FGFR1 in primordial follicular formation. Briefly, neutralizing of FGFR1 leads to a decrease in the number of oocytes (Fig. S9, *A* and *B*). In order to further verify the function of FGF23 and FGFR1, we performed the *in vitro* reconstitution primordial follicle–like structure system. The results showed that the ovaries were cultured for 4 days with neutralizing antibodies and then digested into single cells for reconstruction (Fig. S9*C*). The results showed that neutralizing of FGFR1 or FGF23 blocked the primordial follicular–like structures’ formation efficiency, in which the majority of the cells were aggregated into reconstructed cell masses (Fig. S9, *D*–*F*). All these results preliminarily suggest that FGF23 and FGFR1 function during primordial follicle formation.

To verify the effect of FGFR1 on oocyte meiosis progression *in vitro*, we used synaptonemal complex protein 3 (SYCP3) to mark the meiosis process in the oocytes and performed immunofluorescence costaining to further determine the effect of FGFR1 on oocyte meiosis progression (44, 45). By combining the staining of SYCP3 and DDX4 with a high-resolution confocal imaging system, it showed that the meiotic phases of oocytes were well-identified after the ovaries were cultured with PD173074 from 13.5 dpc for 4 days (equal to 17.5 dpc *in vivo*). Consistent with the previous results, most of oocytes have entered into pachytene and diplotene stages (Fig. S6*C*), and a small portion of germ cells were already arrested at dictyate stage (Fig. S6*C*). Statistical analysis further confirmed that activation of FGFR1 is not essential for oocyte meiosis progression (Fig. S6*D*).

### Apoptosis, rather than autophagy, is involved in massive oocytes death but is not the major cause

To further ascertain whether apoptosis or autophagy was involved in oocytes death correlated to FGF23 signals, we firstly examined the changes of LC3B in our *in vitro* culture model. The level of autophagy in the cultured ovaries treated by FIIN-2 was unaffected, while the apoptosis level indicated by the phosphorylated caspase 3 was significantly elevated, as compared to the control (Fig. 3, *E* and *F*). The results were in line with the RNAi assay (Fig. 2*A*). Therefore, apoptosis, instead of autophagy, is more responsible for explaining why inhibition of FGF23-FGFRs induced significant oocyte loss *in vitro*.

**Figure 5:**
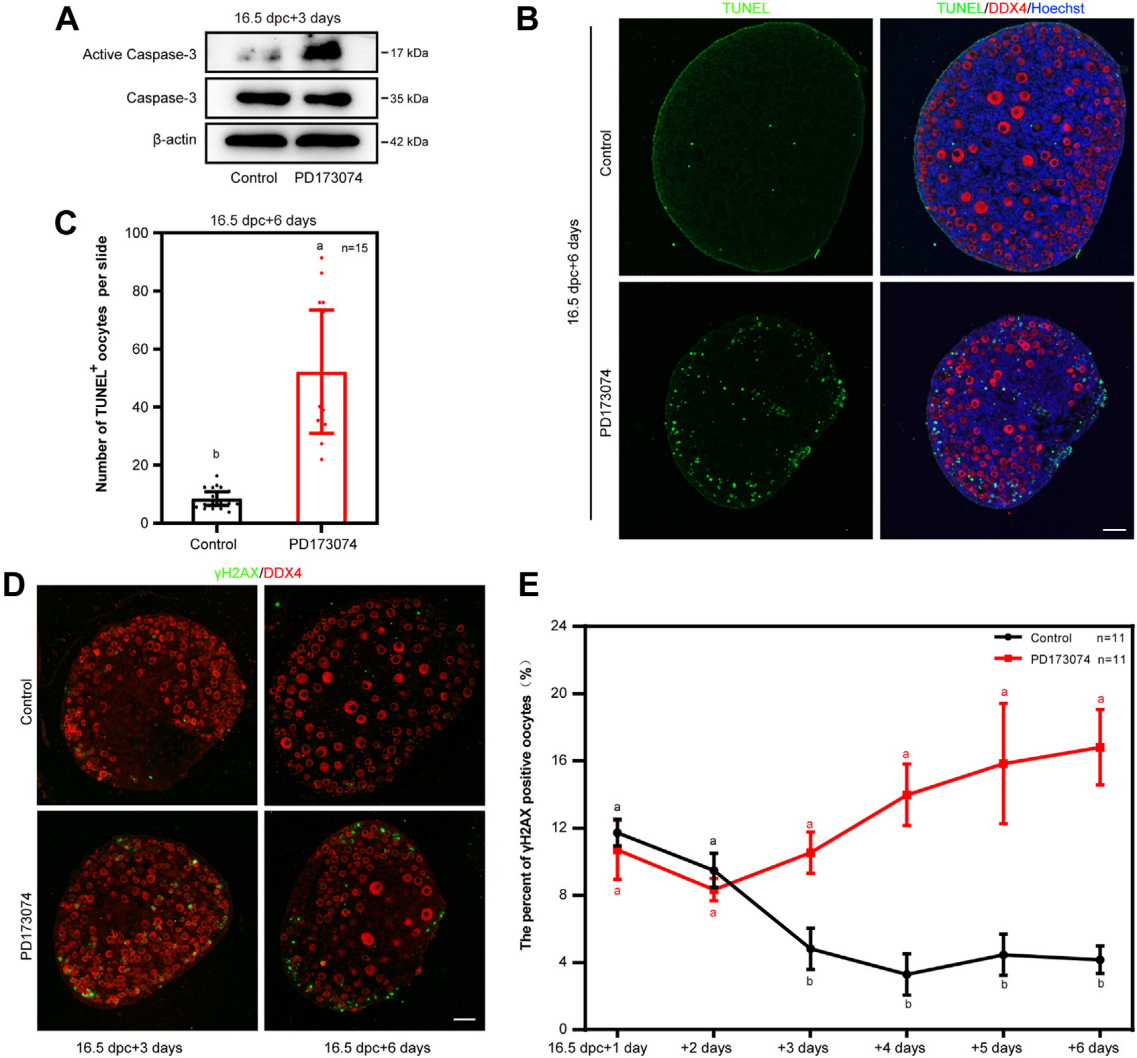
**Specific inhibition of FGFR1 resulted in extensive oocytes apoptosis and oocytes loss.***A*–*C*, inhibition of FGFR1 by PD173074 significantly increased the level of apoptosis. The level of active caspase 3 was obviously higher in PD173074 group than in the control *A*, quantification of TUNEL^+^ oocytes. Oocytes were stained with DDX4 (*red*), TUNEL (*green*), and Hoechst (*blue*). The scale bars represent 50 μm (*B*). The number of TUNEL-positive signals was increased after inhibiting FGFR1. n = 15. Mice ovaries were taken from at least three biologically independent experiments. Data are presented as mean ± SD, and different letters (a, b) indicate significant differences among groups (two-sided ANOVA test), *p* (a, b) < 0.05 (*C*). *D* and *E*, the time-dependent inhibitory effects of PD173074 on the γH2AX^+^ oocytes number. Oocytes were stained with DDX4 (*red*) and γH2AX (*green*). The scale bar represents 50 μm (*D*). The number of TUNEL-positive signals was increased after inhibiting FGFR1. n = 11. Mice ovaries were taken from at least three biologically independent experiments. Data are presented as mean ± SD, and different letters (a, b) indicate significant differences among groups (two-sided ANOVA test), *p* (a, b) < 0.05 (*E*). DDX4, DEAD-box helicase 4; FGFR, fibroblast growth factor receptor.

To explore the reasons for the massive loss of oocytes after inhibiting FGFR1, we performed the following assays. Firstly, the level of active caspase 3, which was located in the oocytes, was obviously higher in PD173074-treated group than in the control (Fig. 5*A*). Secondly, the number of TUNEL-positive signals was increased after inhibiting FGFR1 by PD173074 (Fig. 5, *B* and *C*). Thirdly, the level of the phosphorylated histone H2AX at Ser139 (referred to as γ-H2AX), which marks DNA double-strand breaks (DSBs), was used to test the completeness of the repair of DNA damage. The results showed that the percentage of γ-H2AX signaling increased significantly when 16.5 dpc ovaries were cultured with PD173074 for 6 days. Fourthly, the number of apoptotic cells, as were indicated by both the Annexin Ⅴ– and propidium iodide–positive cells, increased time dependently by the PD173074 treatment *in vitro* (Fig. S10*A*). Further, to test the dynamic changes in the number of positive signals after being treated with PD173074, 16.5 dpc ovaries were cultured for different days. The statistical analysis showed that the signal of positive γ-H2AX became significant since the third day till the sixth day, as compared with the control (Fig. 5, *D* and *E*). When we transfected *Fgf23*-shRNA vectors (shRNA-*Fgf23*) into either KGN cells or KK1 cells and cultured the cells for 2 days, the cell viability in both the assays was decreased as well (Fig. S10, *B*–*D*). Together with the findings in Figure 5, it is assumed that FGF23 in granulosa cells is most possibly involved in the regulation of granulosa cells apoptosis.

Subsequently, to determine whether apoptosis was the main cause for oocyte loss after FGF23 was blocked, Z-VAD-FMK, a pan-caspase inhibitor, was added to the culture media of 16.5 dpc ovaries with PD173074 and cocultured for 6 days. The results showed that the number of oocytes was not significantly changed (Fig. S10, *E* and *F*). Therefore, inhibition of FGFR1 eventually leaded to apoptosis of germ cells, but apoptosis was not the cause of mass loss of germ cells.

### FGFR1 prevents oocytes loss through inhibiting MAPK signaling pathway

**Figure 6:**
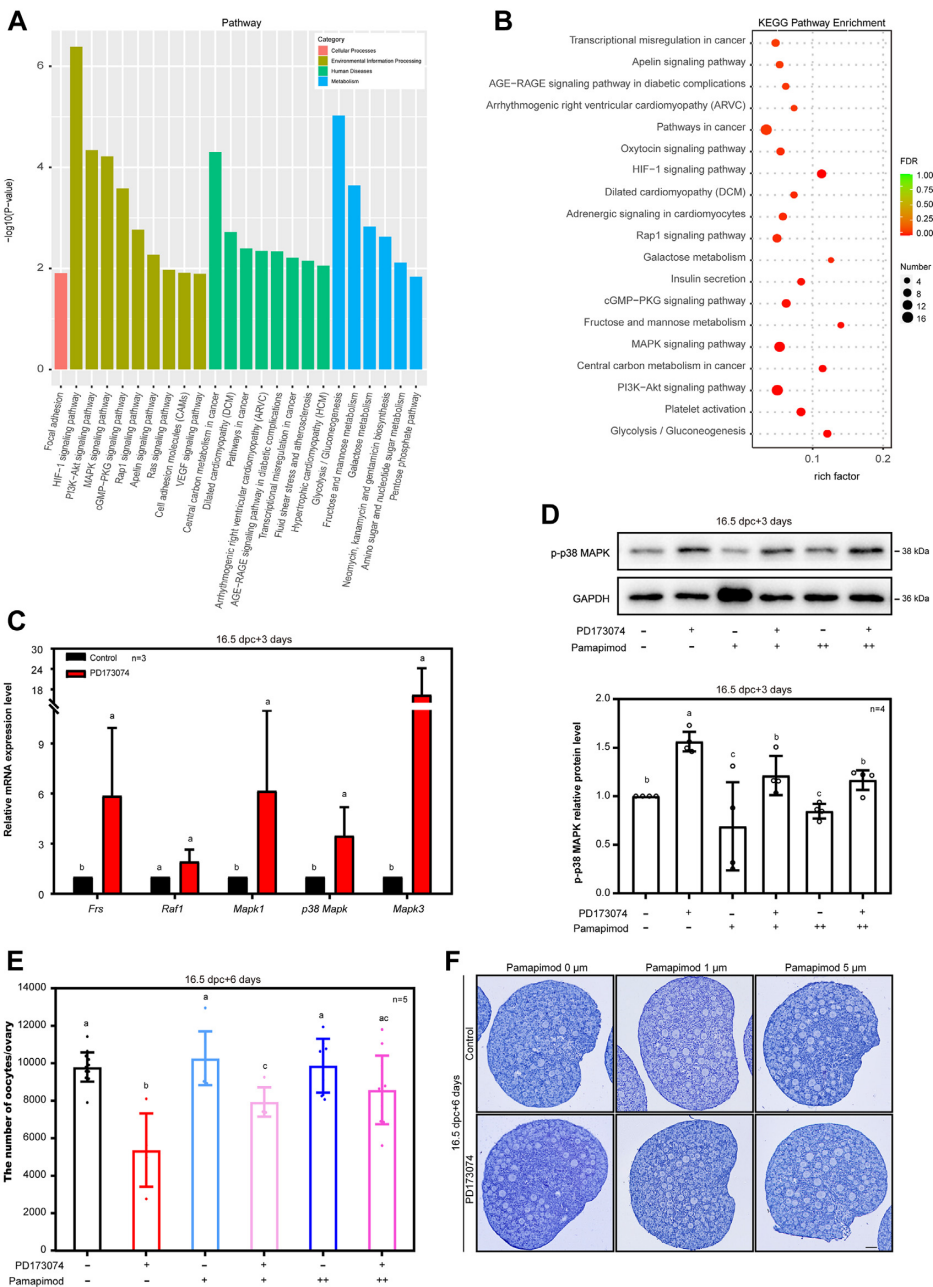
**The p38 MAPK signaling pathway in the oocytes was one of the key downstream responsive molecules of FGF23 during primordial follicle formation.***A* and *B*, preliminary analysis of RNA-seq data showed that the changes in cellular adhesion and MAPK signaling pathways were significant. *C*, the mRNA expressions of MAPK signaling pathway after inhibition of FGFR1 in mouse ovaries were assessed by qRT-PCR. *D*–*F*, the rescue of oocytes number by Pamapimod in PD173074-treated ovaries. n = 4 biologically independent experiments. Data are presented as mean ± SD, and different letters (a–c) indicate significant differences among groups (two-sided ANOVA test), *p* (a, b) < 0.05, *p* (a, c) < 0.05, *p* (b, c) < 0.05 (*D*). Quantification of oocytes was carried out in the control and the PD173074-treated ovaries. n = 5. Mice ovaries were taken from at least three biologically independent experiments. Data are presented as mean ± SD, and different letters (a–c) indicate significant differences among groups (two-sided ANOVA test), *p* (a, b) < 0.05, *p* (a, c) < 0.05, *p* (b, c) < 0.05 (*E*). Inhibition of p38 MAPK effectively rescued the oocyte loss caused by PD173074. The scale bars represent 50 μm (*F*). FGF, fibroblast growth factor; FGFR, fibroblast growth factor receptor; MAPK, mitogen-activated protein kinase; qRT-PCR, quantitative reverse transcription-PCR.

To explore the intracellular mechanism of the FGF23-FGFR1 signaling in regulating oocyte apoptosis, 16.5 dpc ovaries were cultured with or without PD173074 for 3 days *in vitro*. The preliminary analysis of RNA-seq data indicated significant changes in cellular adhesion and MAPK signaling pathways induced by PD173074 (Fig. 6, *A* and *B*). In line with these findings, most of the mRNA levels of the MAPK signaling pathway–related molecules were significantly upregulated after the 16.5 dpc ovaries were cultured for 3 days (Fig. 6*C*). Furthermore, phosphorylated p38 MAPK (p-p38 MAPK) levels were significantly upregulated, as compared to the control (Fig. 6*D*). However, Western blotting analyses revealed that after inhibition of FGFR1, other proteins, such as p62, AKT/p-AKT, and mTOR/p-mTOR, did not change significantly (Fig. S11).

To verify whether p38 MAPK signaling pathway was the key to germ cells depletion, pamapimod, one of the p38 MAPK specific inhibitors, was added to the culture media of 16.5 dpc ovaries. The results indicated that the p-p38 MAPK level was significantly decreased by either 1 μM or 5 μM of pamapimod (Figs. S12 and 6*D*). When 16.5 dpc ovaries were cultured for 6 days, more oocytes were observed in PD173074 plus pamapimod treatment than those in the PD173074 treatment alone (Fig. 6, *E* and *F*). Therefore, specific inhibition of p-p38 MAPK reversed the mass loss of oocytes induced by PD173074.

## Discussion

In most mammals, programmed loss of oocytes occurs briefly around the time of birth, but the reasons and the mechanisms remain uncertain (5, 46). Cellular apoptosis is the first recognized process responsible for oocyte PCD (6, 47, 48). This is not only because low-level apoptosis occurs before birth but because increase or decrease in follicle numbers occurred in postnatal mice bearing systemic deletions of proapoptotic or antiapoptotic genes (49). Most recently, we have proved that GSK-3β is essential for sustaining fetal oocyte survival by fine-tuning the cytoplasmic-nuclear translocation of β-catenin, which in turn modulates TAp63 expression timely during meiotic prophase I mice promptly (7). However, it is unknown what kinds of extracellular molecules participate in oocyte apoptosis during primordial follicle formation. Here, we have proved that FGF23 as a pregranulosa cell–derived paracrine factor prevents premature oocyte apoptosis and protecting oocytes from massive loss at physiological condition.

During primordial follicle formation, the granulosa cells gradually envelop the dominant oocyte. During this process, many paracrine or endocrine factors are involved to ensure proper assembly of the primordial follicle. For example, maternal progesterone levels in midpregnancy inhibit the expression of *Jagged2* and *Notch1* in mouse fetal mouse, which markedly inhibited nest breakdown and follicle formation (50). In addition, the interaction of granulosa cell–derived KitL with oocyte- and membrane-derived Kit has an important role in the migration and proliferation of primordial germ cells and follicle development. All these studies showed that the close communication between granulosa cells and oocytes is important for primordial follicle formation (51, 52, 53). The results from this study emphasized the importance in the role of granulosa cells–drove paracrine factor FGF23 in protecting oocytes from premature loss in primordial follicle formation in mice. *In vitro*, impaired function of FGF23 before primordial follicle formation leads to activated MAPK signal pathway in mice oocytes, which induces significant oocyte apoptosis in ovaries, implying that FGF23 is pivotal for supporting DSB repair completion. Although our results suggest that the mutual spatiotemporal expression patterns of FGF23 and FGFR1 might be closely related to the PCD of oocytes in the mouse ovary around the time of birth, the underlying mechanism that coordinates the substantially programmed meiotic DSB existence and the DNA damage checkpoint in fetal oocytes is still elusive.

This study has proved that at least one of the FGFRs participates in mediating the function of FGF23 during primordial follicle formation *in vitro*. Previous studies have shown that intact FGF23 signaling activates FRS2/RAS/RAF/MEK/ERK1/2 (54, 55). The results of our study have shown that the mRNA levels of FRS2/RAS/ERK1/2 were changed systematically (Fig. 6*C*), but the level of the protein expression and the activity (phosphorylation) of ERK1/2 did not change significantly. In contrast, in mouse ovaries, FGF23 activates P38 through activating, at least FGFR1, and plays a pivotal role in supporting primordial follicle formation. In addition, we noticed no significant changes in p62 protein level after knockdown of *Fgf23*, no changes in either lysine-specific demethylase or p62 protein levels, and no changes in relative mRNA expression level of GSK-3β after FGFRs inhibition (Fig. S13). This also provides preliminary evidence that the FGF23–FGFR1 pathway is different from the pathway previously studied, which also provides a new mechanism for regulating oocyte number during primordial follicle formation. Whether there are other signaling pathways that are pivotal for conducting FGF23 remains unclear.

Despite these, plenty of works are needed to provide substantial data that are helpful to give precise explanations upon the FGF23-involved oocyte fate determination in mice. For instances, although this study highlights that FGF23 mainly binds to FGFR1 to protect oocytes from mass loss, the specific roles of FGFR2, FGFR3, and FGFR4 have not been excluded (56). Due to the high homology of FGFR1-FGFR4, the knockdown effect of single receptor constructed in this study needs optimization, and more detailed experiments, including *in vivo* assays, are needed for further verification. Moreover, the function of the FGF23 in mice ovaries is obviously different from the conventionally reported so far, it is therefore necessary to further explore if the metabolism of the mineral particles, such as Na^+^, Ca^2+^ and phosphorus, are affected by the FGF23 in the ovaries (57, 58). Lastly, although the follicle developmental, stage-depended roles of FGF23 was not systematically studied here, the expression patterns of FGFRs suggested that FGF23 might be active in affecting the fate of the growing follicles as well (results not shown). Hence, the physiological roles of FGF23 in adult ovaries need further study through constructing conditionally modified animals in the future (59).

## Conclusion

**Figure 7:**
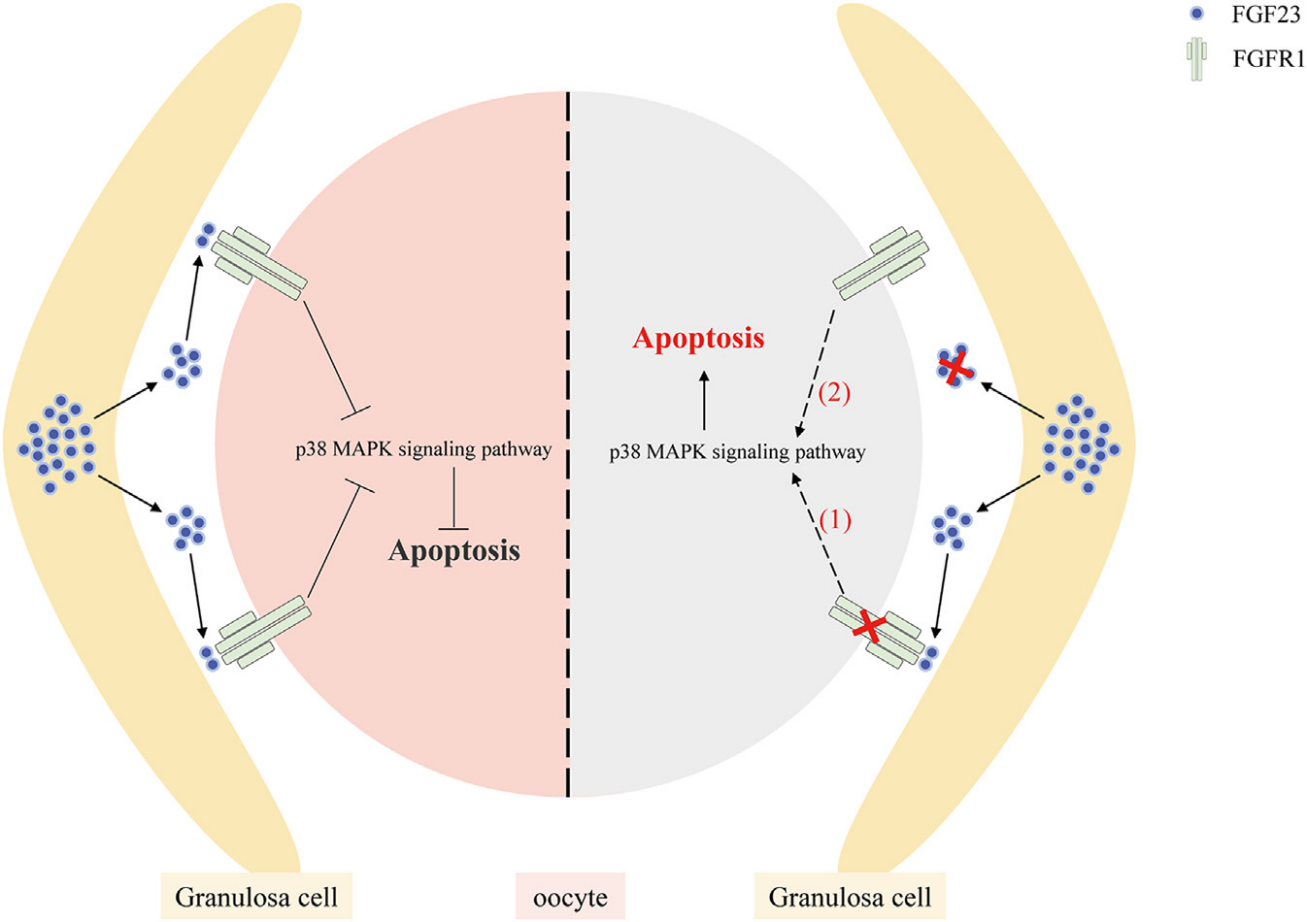
**A proposed model: FGF23-FGFR1 regulates apoptosis to protect oocytes from premature loss during primordial follicle formation.** By inhibiting p38 MAPK signaling pathway in the oocytes, FGF23-FGFR1 plays an indispensable role in protecting mouse oocytes from accumulation of DSBs, which leads to apoptosis at the stage of primordial follicle formation. DSB, double-strand break; FGF, fibroblast growth factor; FGFR, fibroblast growth factor receptor; MAPK, mitogen-activated protein kinase.

We approved here that pregranulosa cell–secreted FGF23 prevents oocyte from premature apoptosis through activation of the p38 MAPK pathway downstream of FGFR1 in perinatal mice ovaries (Fig. 7). This study emphasizes not only the importance of the mutual communication between the germ cells and the OSCs during primordial follicle formation but is helpful to explain the primordial follicle size-determining mechanisms that are pivotal for understanding the physiological regulation of primordial follicle formation.

## Experimental procedures

### Animals

CD1(ICR) mice were purchased from Beijing Vital River Laboratory Animal Technology Co, Ltd. Mice were housed under controlled lighting (12 h light and 12 h dark) and temperature (22–26 °C) conditions with free access to food and water. Female mice were mated with males overnight and checked for a vaginal plug the following morning. The presence of a vaginal plug was considered 0.5 dpc. The day after partum was considered to be 1 dpp. All procedures were conducted in accordance with the guidelines of and approved by the Animal Research Committee of the China Agricultural University.

### Ovary isolation and culture

The mice were sacrificed at the designated time. Ovaries were separated by microdissection from the mesonephros or ovarian capsule in prechilled PBS (10 mM, pH 7.4) under a stereomicroscope (ZSA302, COIC). The isolated ovaries were cultured in 6-well culture dishes (NEST Biotechnology) with 1.2 ml of Dulbecco’s modified Eagle’s medium/Ham F12 nutrient mixture (Gibco, Life Technologies) with 1% penicillin-streptomycin solution at 37 °C, 5% CO_2_, 95% air atmosphere with saturated humidity. Ovaries were cultured in 6-well culture plates (NEST) in either medium with dimethylsulfoxide or medium supplemented with inhibitor. The culture medium was exchanged every other day. The final concentration of FIIN-2 (S7714, Selleck), PD173074 (S1264, Selleck), and FGF23(30R-AF040, PeproTech) were 0.25 μM, 4 nM, and 1 to 500 μM, respectively.

### Plasmid construction and RNAi

To knockdown the *Fgf23* gene, shRNA*-Fgf23* was cloned into pSicoR-GFP. Golden star t6 super PCR mix was purchased from Tsingke Co, Ltd. All of the constructs were verified by sequencing. Primers are listed in Table S1.

To assure that siRNA vectors would be transfected into the inner cells of fetal ovaries, the siRNAs vectors were injected into isolated 16.5 dpc ovaries using glass pipettes with a stereomicroscope. After the ovaries were full of liquid, electrotransfection (ECM2001, BTX) was performed by applying three 5-ms-long quasi square pulses at a pulse-field strength of up to 30 V/cm. Ovaries were cultured for 72 h to test the transfection efficiency of protein levels or for 6 days for histological examination and oocyte counting.

### Immunofluorescence

Ovaries were fixed in 4% paraformaldehyde overnight, embedded in paraffin, and sectioned at 5 μm. After dewaxing, rehydration, and high-temperature (96–98 °C) antigen retrieval with 0.01% sodium citrate buffer (pH 6.0), the sections were blocked with 10% normal serum for at least 60 min at room temperature and immunostained with primary antibodies for 12 h at 4 °C. Subsequently, after rinsing thoroughly with PBS, the slides were then incubated with fluorophore-conjugated secondary antibodies (Alexa Fluor 488– or Alexa Fluor 555–conjugated donkey secondary antibodies against mouse, rabbit, and goat IgG) (1:200; Invitrogen) and Hoechst 33342 (1:1000; B2261, Sigma) at 37 °C for 1 h. Slides were then rinsed in PBS and sealed in antifade fluorescence mounting medium (Applygen) with coverslips. Sections were examined and photographed using Nikon Eclipse 80i digital fluorescence microscope or Nikon A1 laser scanning confocal microscope. Primary antibodies were listed in Table S2.

### Histological sections and follicle counts

Ovaries were fixed in 4% paraformaldehyde overnight, embedded in paraffin, and serially sectioned at 5 μm. The ovarian sections were stained with hematoxylin or Alexa Fluor 488, the number of follicles per ovary was counted in every fifth section. To estimate the total numbers of oocytes in each ovary, the sum was multiplied by five.

### Isolation of granulosa cells and oocytes

Mouse ovaries were collected in 1.5 ml tubes and incubated with 0.2 ml of 0.25% trypsin at 37 °C for 2 min and repeated four times. During each incubation, the ovaries were pipetted up and down to digest them into single cell suspensions. A total of 0.2 ml fetal bovine serum was added to each tube to terminate the digestion reaction. The supernatant was removed by centrifugation, and the precipitate was resuspended in 1.2 ml of Dulbecco’s modified Eagle’s medium/Ham F12 nutrient mixture with 10% fetal bovine serum and 1% insulin-transferrin-selenium solution (51500056, Life Technologies). The cell suspension was transferred into 6-well plates and cultured at 37 °C, 5% CO2, and 95% air atmosphere with saturated humidity for 4 to 5 h. The culture medium was collected and centrifuged to collect the oocytes. The adherent cells (somatic cells) were digested with trypsin and collected after centrifugation.

### Western blotting analysis

Each protein sample were extracted in tissue and cell lysis reagent (for ovaries) or radio immunoprecipitation assay (for cells) tissue and cell lysis solution containing 1% PMSF (Cell Signaling Technologies) according to the manufacturer's instructions. Samples were separated on 10% SDS-PAGE gels and then transferred onto methanol-pretreated polyvinylidene fluoride membranes (IPVH00010, Millipore). SDS-PAGE and immunoblots were performed following standard procedures using a Mini-PROTEAN Tetra Cell System (Bio-Rad). Membranes were blocked with 5% skim milk powder for at least 1 h at room temperature and then incubated with appropriate primary antibodies for 8 to 12 h at 4 °C. Then, membranes were incubated with the secondary antibody (1:5000, ZSGB-BIO) for 1 h at room temperature and rinsed with tris buffered saline with tween 20. The membranes were visualized using a SuperSignal West Pico Chemiluminescent Detection System (Prod 34080, Thermo Fisher Scientific). The antibodies used are listed in Table S2.

### RNA isolation and real-time RT-PCR

Ovaries in each group were extracted by TRIZOL Reagent (Invitrogen, Life Technologies). One microgram total RNA was used to synthesize complementary DNA according to manufacturer’s instructions (Promega Reverse Transcription System, Promega). qRT-PCR was operated in a Power SYBR Green PCR Master Mix (Applied Biosystems, Life Technologies) and analyzed by Applied Biosystems 7300 Real-Time PCR System (Life Technologies). Primers were listed in Table S1.

### *In situ* karyotyping

Generally, after dewaxing, rehydration, and high-temperature (96–98 °C) antigen retrieval with 0.01% sodium citrate buffer (pH 6.0), the paraffin sections (thickness, 5 μm) of the ovaries were blocked with 10% normal serum for at least 60 min at room temperature and incubated with SYCP3 antibody and DDX4 antibody at 4 °C overnight (more than 16 h) to label the synaptonemal complex and distinguish the meiotic stages of germ cells. Subsequently, after rinsing thoroughly with PBS, the slides were then incubated with fluorophore-conjugated secondary antibodies (Alexa Fluor 488– or Alexa Fluor 555–conjugated donkey secondary antibodies against mouse, rabbit IgG) (1:200; Invitrogen) at 37 °C for 1 h. And then counterstained by Hoechst 33342 (1:1000) to identify the nucleus. Slides were then rinsed in PBS and sealed in antifade fluorescence mounting medium (Applygen) with coverslips. Sections were examined and photographed using Nikon Eclipse 80i digital fluorescence microscope or Nikon A1 laser scanning confocal microscope.

The detail indexes of identification are listed here: leptotene, abundant chromatin fibrils appear in nucleus; zygotene, chromosome, and classical tripartite synaptonemal complex structure are clearly visible; and pachytene, the chromosomes are the shortest and thickest. Homologous chromosome association and homologous recombination are completed; diplotene, the homologous chromosomes begin to separate from each other; dictyate, the chromosomes are decondensed and diffused; and two to four bright spots appear in the nucleus.

### Statistical analysis

Each experiment was repeated at least three times. The data were analyzed by two-tailed unpaired ANOVA. A *p* value of less than 0.05 was considered statistically significant. The data are shown as the mean ± SD.

## Data availability

The data that support the findings of this study are available from the corresponding author upon reasonable request.

## Supporting information

This article contains supporting information.

## Ethics statement

All animal procedures were conducted in accordance with the guidelines of and approved by the Animal Research Committee of the China Agricultural University.

## Conflict of interest

The authors declare that they have no conflicts of interest with the contents of this article.
