# Supplementary material for: Pregranulosa cell–derived FGF23 protects oocytes from premature apoptosis during primordial follicle formation by inhibiting p38 MAPK in mice
Source: J Biol Chem. 2023 May 2;299(6):104776. doi: 10.1016/j.jbc.2023.104776 (PMC10240423; doi:10.1016/j.jbc.2023.104776)
Supplement: Supporting Figures S1–S13 and Tables S1 and S2 [file mmc1.docx]

**Supplementary data**


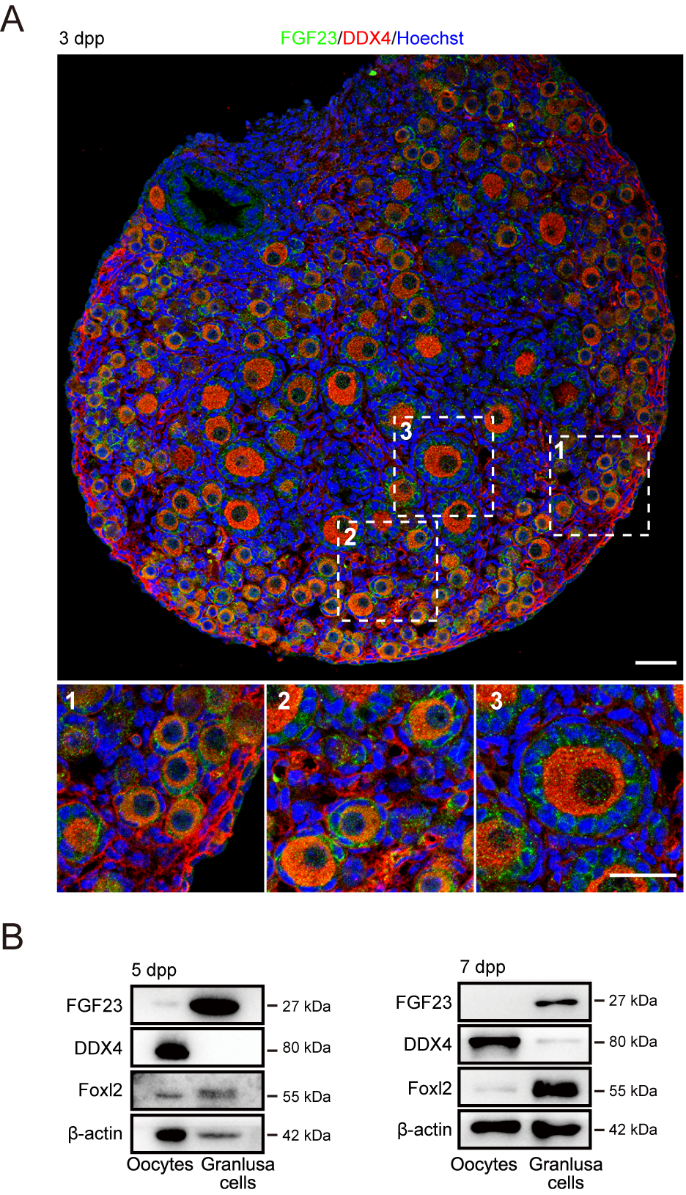


**Fig. S1 The expression pattern of FGF23 in ovaries of new born mice.**

**A** Cellular localization of FGF23 in mouse ovaries. Newborn mouse ovaries were stained for FGF23 (green) and the oocyte marker DDX4 (red) at 3 dpp. Locally enlarged area of the “box” in the image above were shown to present the more detailed follicular structure. The nuclei were dyed with a Hoechst counterstain (blue). FGF23 was mainly localized to the cytoplasm of granulosa cells in either primordial follicles or growing follicles. Scale bars: 50 μm. **B** Localization of FGF23 in mouse ovaries. FGF23 was mainly localized to the granulosa cells.


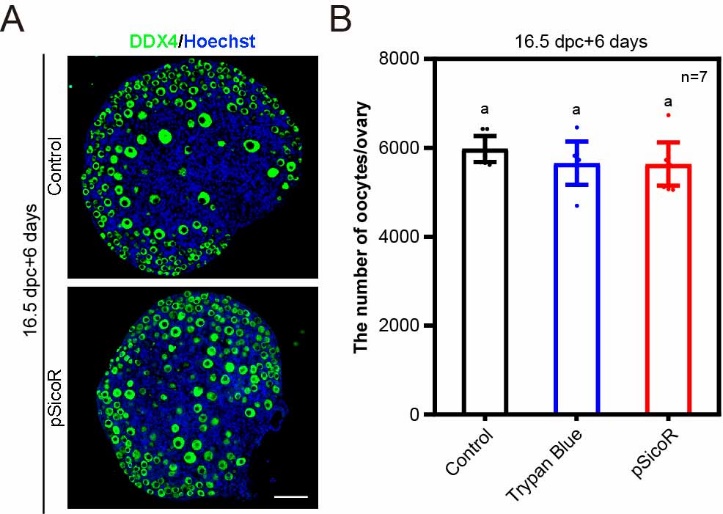


**Fig. S2 The knockdown model was successfully established *in vitro*.**

**A, B** The histological and statistical analysis results showed that the numbers of germ cells was consistent with the untreated or injected trypan blue ovaries. Scale bars: 50 μm. n=7. Mouse ovaries were taken from at least 3 biologically independent experiments. Data are presented as mean ± SD (two-sided ANOVA test).


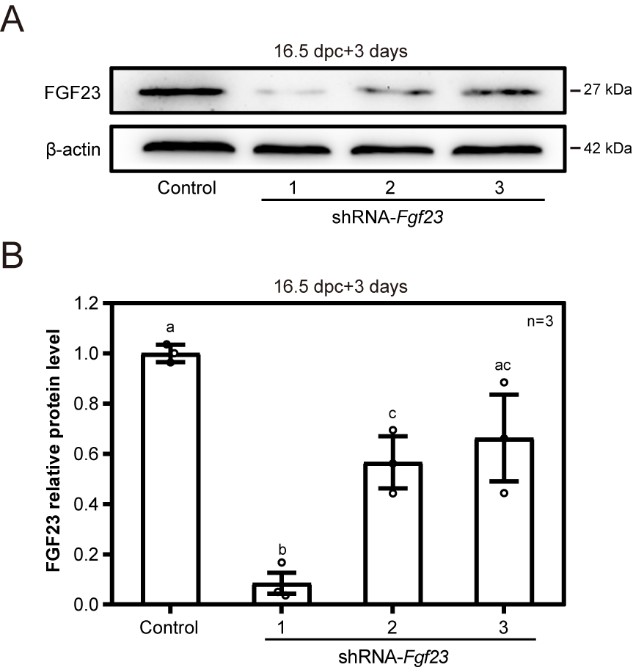


**Fig. S3 Knockdown efficiency analysis of shRNA-*Fgf23*.**

**A, B** FGF23 protein level was significantly reduced in shRNA-*Fgf23* group ovaries compared to the control after 3 days of culture. Among the three shRNAs of *Fgf23*, the efficiency of shRNA-*Fgf23*-1 was the highest. n=3 biologically independent experiments. Data are presented as mean ± SD, and different letters (a-c) indicate significant differences among groups (two-sided ANOVA test), P (a, b) < 0.05, P (a, c) < 0.05, P (b, c) < 0.05.


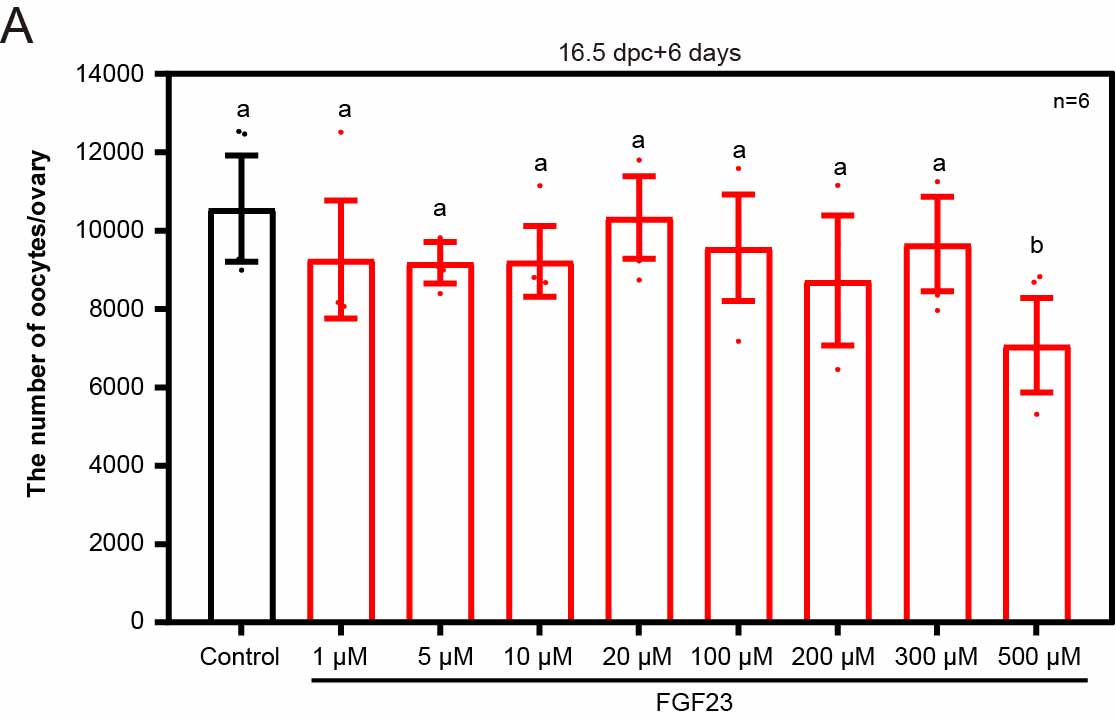


**Fig. S4 Excess FGF23 supplementation had no significant effect on the number of oocytes.**

**A** The follicle counting results showed that superfluous FGF23 did not affect the number of total follicles. n=6. Mice ovaries were taken from at least 3 biologically independent experiments. Data are presented as mean ± SD.


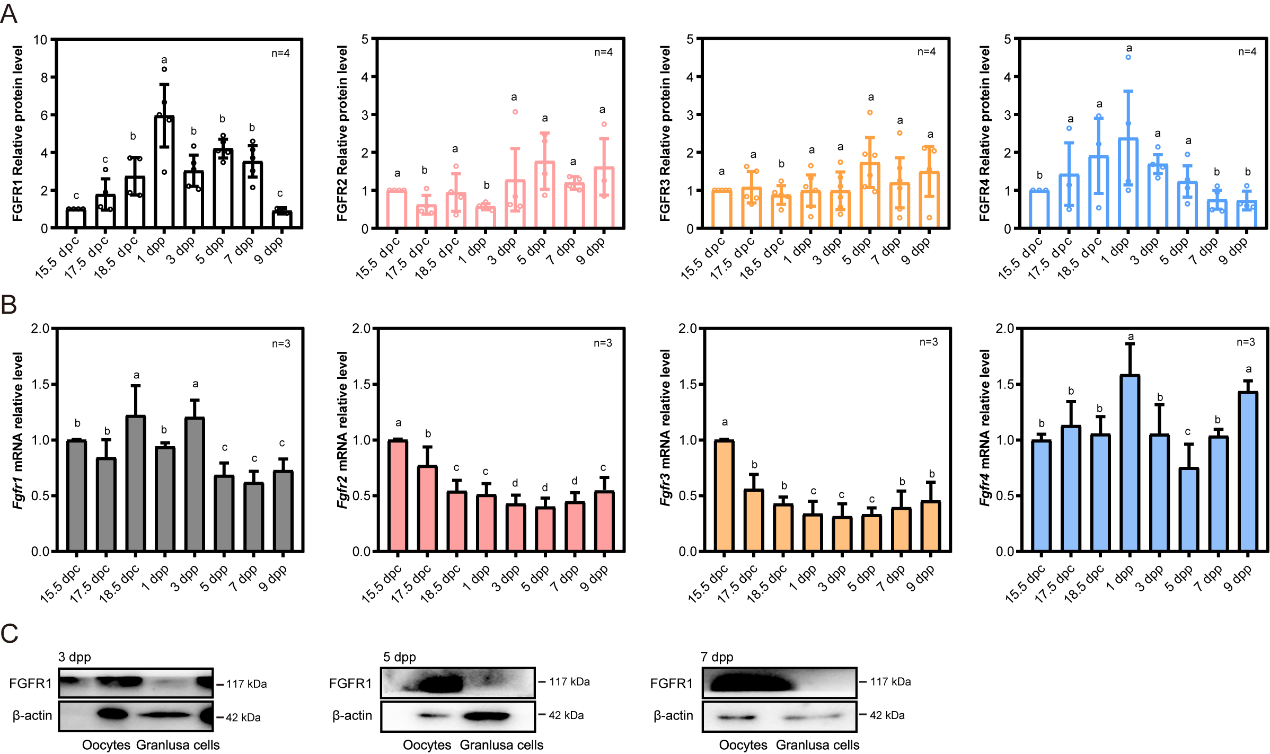


**Fig. S5 The expression patterns of FGFRs in mouse ovaries.**

**A** The protein expressions of FGFRs were determined by Western blotting with β-actin as a loading control from 15.5 dpc to 9 dpp. n=4 biologically independent experiments. Data are presented as mean ± SD, and different letters (a-c) indicate significant differences among groups (two-sided ANOVA test), P (a, b) < 0.05, P (a, c) < 0.05, P (b, c) < 0.05. **B** The mRNA expression levels of *Fgfrs* were assessed using qRT-PCR and normalized to β-actin. n=3 biologically independent experiments. Data are presented as mean ± SD, and different letters (a-d) indicate significant differences among groups (two-sided ANOVA test), P (a, b) < 0.05, P (a, c) < 0.05, P (a, d) < 0.05, P (b, c) < 0.05, P (b, d) < 0.05, P (d, c) < 0.05. **C** Localization of FGFR1 in mouse ovaries. FGFR1 was mainly localized to the oocytes.


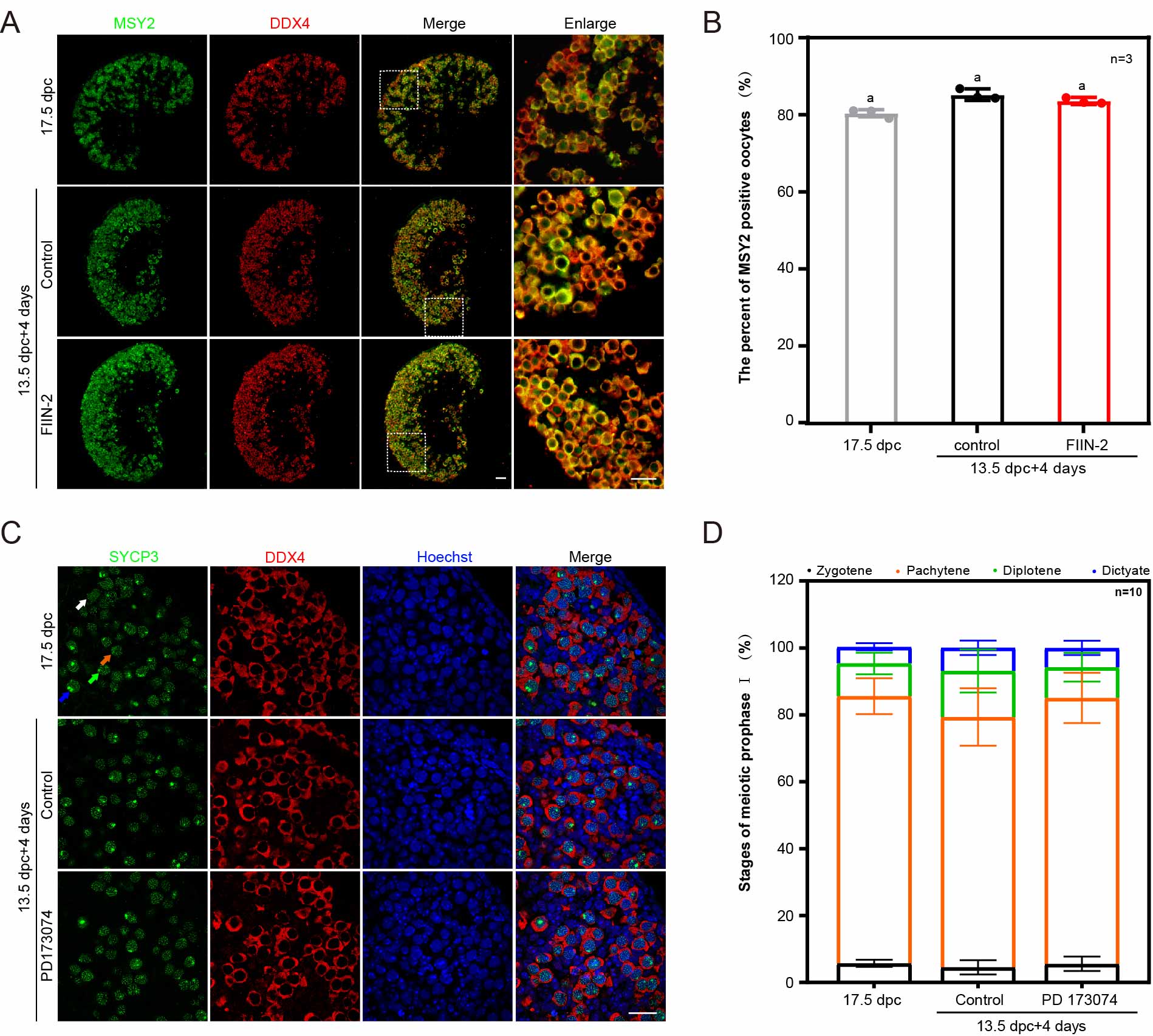


**Fig. S6 FGFRs did not affect the oocyte meiosis process.**

**A, B** The ratio of colocation expression of MSY2 (green) and DDX4 (red) in the FIIN-2 group and the control was unaffected. n=3 biologically independent experiments. Data are presented as mean ± SD (two-sided ANOVA test). Scale bars: 50 μm. **C, D** Most of oocytes have entered into pachytene and diplotene stages (**C**, pachytene, the orange arrow; diplotene, the green arrow), and a small portion of germ cells were even arrested at dictyate stage (**C**, the blue arrow). Scale bars: 50 μm. Statistical analysis confirmed that FGFR1 is not essential for oocyte meiosis. n=10. Mice ovaries were taken from at least 3 biologically independent experiments. Data are presented as mean ± SD (two-sided ANOVA test) (**D**).


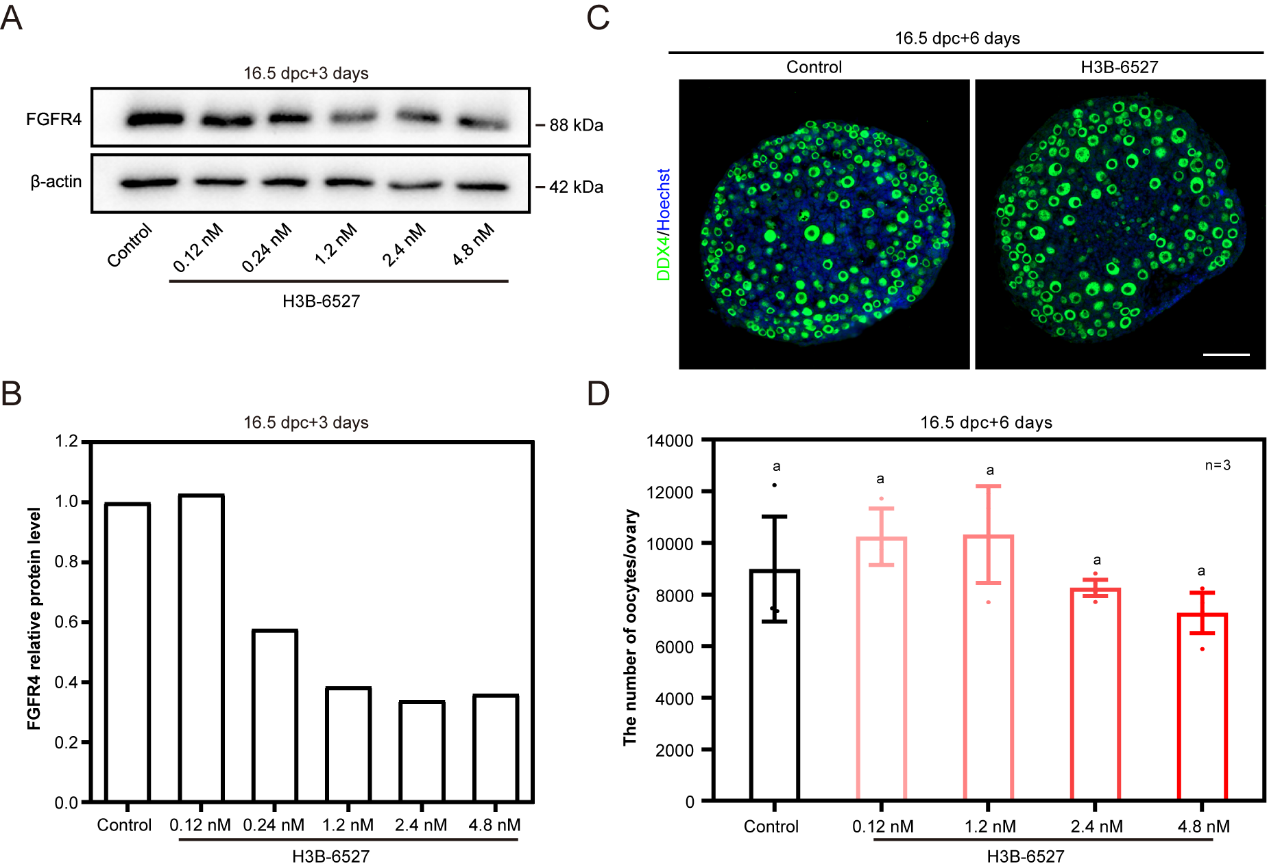


**Fig. S7 FGFR4 is not essential for protecting oocytes from loss during primordial follicle formation.**

**A, B** H3B-6527 significantly suppressed the expression of FGFR4 in cultured ovaries. **C, D** The follicle counting results showed that decreasing FGFR4 did not affect the number of total follicles. Scale bars: 50 μm. n=3 biologically independent experiments. Data are presented as mean ± SD (two-sided ANOVA test).


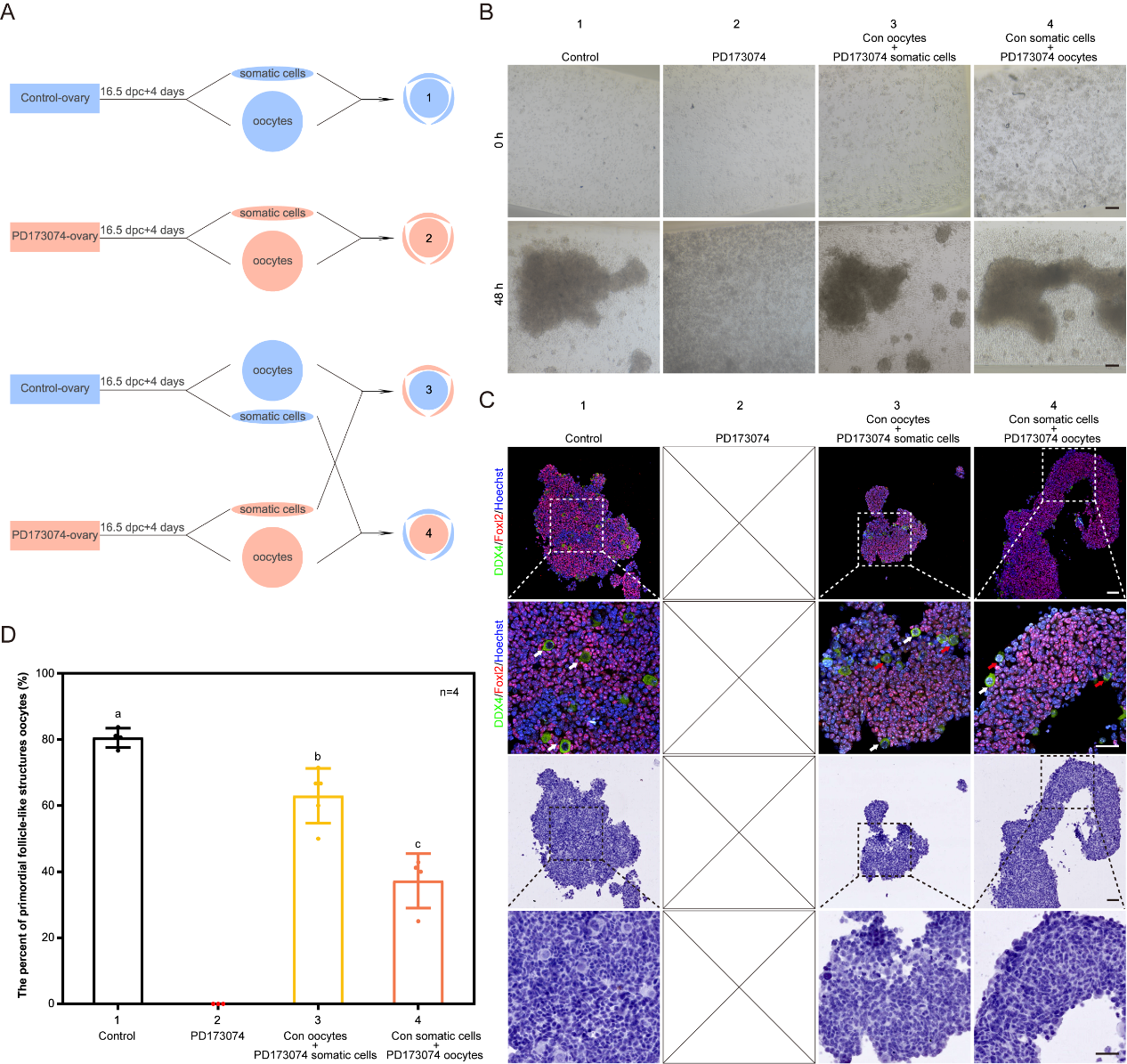


**Fig. S8 Primordial follicles formation were affected by FGFR1.**

**A** Ovaries at 16.5 dpc were cultured for 4 days, and the ovarian cells were dispersed and reconstituted into follicle-like structures (group 1 and 2). Before reconstitution, oocytes and somatic cells derived from ovaries pretreated with or without PD173074 (groups 3 and 4) were exchanged. **B** The tissue structures of the reconstructed cell masses. Scale bar: 500 μm. **C** The tissue structures of the reconstructed cell masses. The follicular structures in the “boxes” in the 1st and 3rd rows of images were enlarged accordingly. DDX4: green, Foxl2: red, Hoechst: blue. White arrows: primordial follicle-like structures. Red arrowheads: granulosa cells failed to enclose oocytes. Scale bar: 50 μm. **D** Quantification of primordial follicle-like structures in the control and the neutralizing antibody-treated ovaries. n=4. Mice ovaries were taken from at least 3 biologically independent experiments. Data are presented as mean ± SD, and different letters (a-c) indicate significant differences among groups (two-sided ANOVA test), P (a, b) < 0.05, P (a, c) < 0.05, P (b, c) < 0.05.


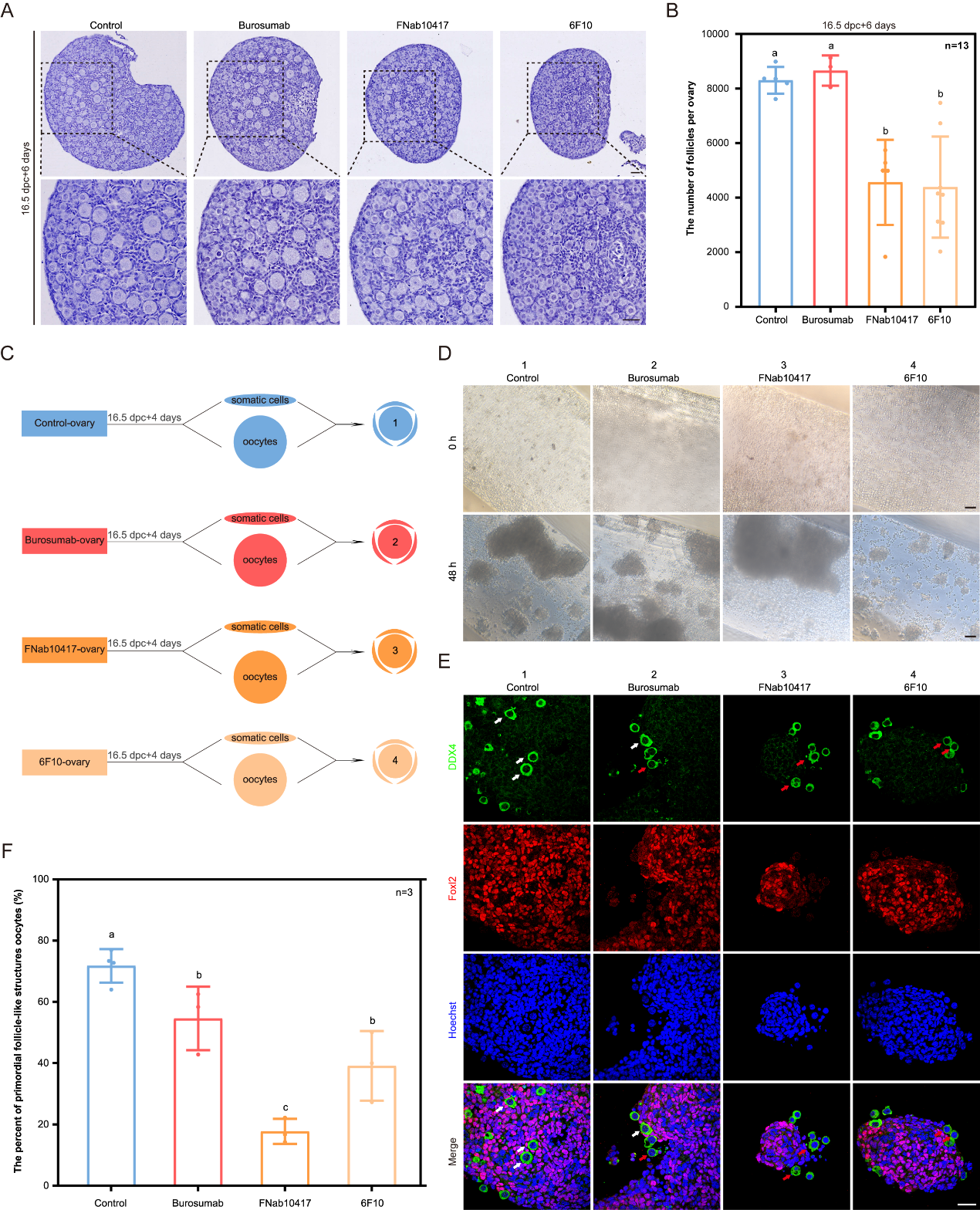


**Fig. S9 Primordial follicles formation was affected by FGF23 and FGFR1 signaling.**

**A, B** Quantification of oocytes in the control and the neutralizing antibody-treated ovaries. The follicular structures of the “boxes” in the image were enlarged to view the detailed information in (A). n=13. Mice ovaries were taken from at least 3 biologically independent experiments. Data are presented as mean ± SD, and different letters (a, b) indicate significant differences among groups (two-sided ANOVA test), P (a, b) < 0.05. Scale bars: 50 μm. **C** Ovaries at 16.5 dpc were cultured for 4 days, and the ovarian cells were dispersed and reconstituted into primordial follicle-like structures (group 1-4). **D** The tissue structures of the reconstructed cell masses. Scale bar: 500 μm. **E** The tissue structures of the reconstructed cell masses. DDX4: green, Foxl2: red, Hoechst: blue. White arrows: follicle-like structures. Red arrowheads: granulosa cells failed to enclose oocytes. Scale bar: 50 μm. **F** Quantification of follicle-like in the control and the neutralizing antibody-treated ovaries. n=3. Mice ovaries were taken from at least 3 biologically independent experiments. Data are presented as mean ± SD, and different letters (a-c) indicate significant differences among groups (two-sided ANOVA test), P (a, b) < 0.05, P (a, c) < 0.05, P (b, c) < 0.05.


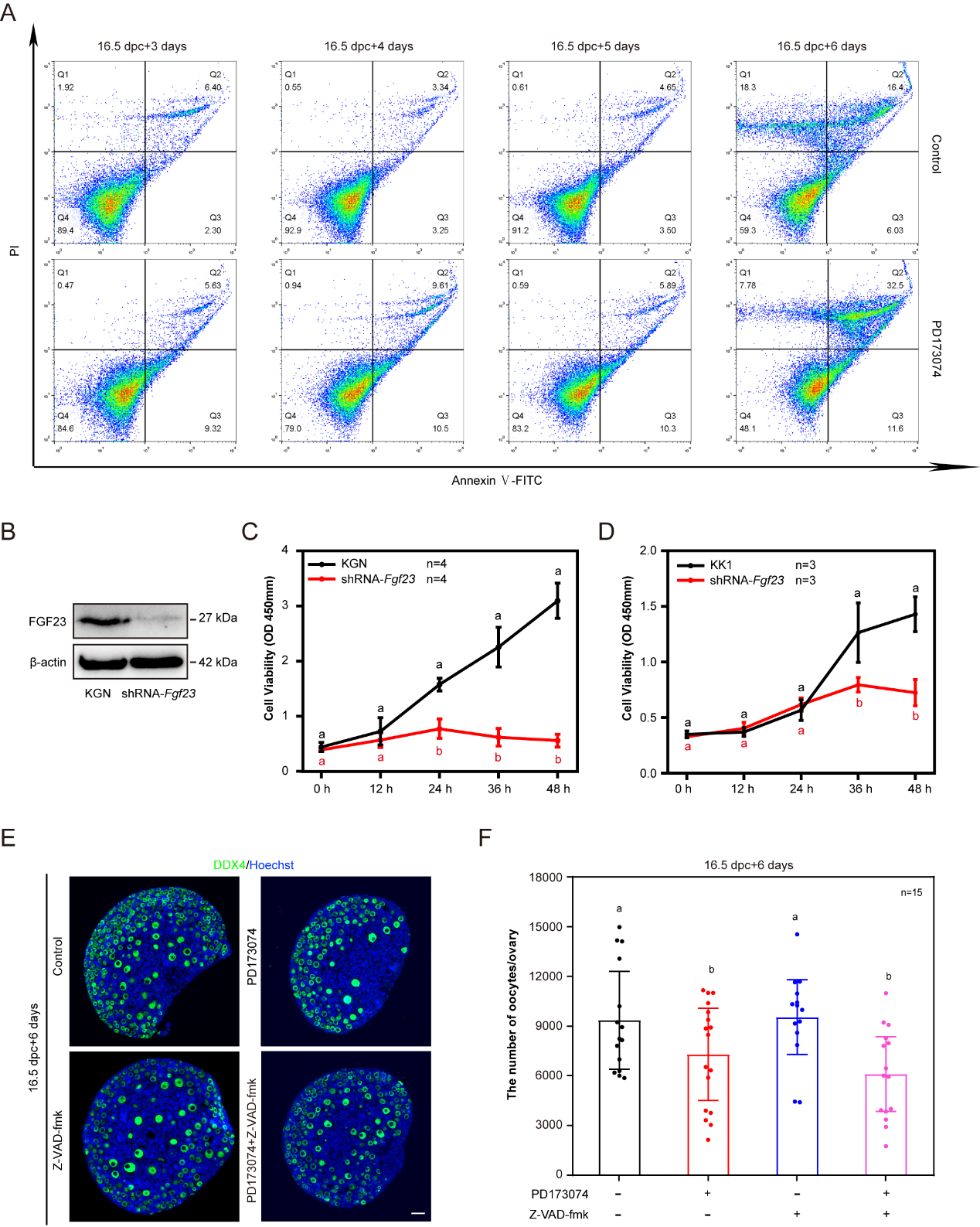


**Fig. S10 Apoptosis was not the cause of mass loss of germ cells.**

**A** Measurement of cell death by Annexin V binding and PI uptake assays. The individual populations can be defined using quadrant gates, which automatically quantify the number of cells in each quadrant as follows: Q1 = nuclei without plasma membrane = (PI^+ve^/Annexin V^−ve^); Q2 = necrotic cells = (PI^+ve^/Annexin V^+ve^); Q3 = apoptotic cells (PI^−ve^/Annexin V^+ve^); Q4 = live cells (PI^−ve^/Annexin V^−ve^).  A quantitative analysis of the rate of apoptosis was performed. **B** FGF23 protein levels were clearly lower in shRNA-*Fgf23* than in the controls after 2 days of culture. **C** *Fgf23* knockdown decreased the cell viability of KGN cells. n=4 biologically independent experiments. Data are presented as mean ± SD, and different letters (a, b) indicate significant differences among groups (two-sided ANOVA test), P (a, b) < 0.05. **D** *Fgf23* knockdown decreased the cell viability of KK1 cells. n=3 biologically independent experiments. Data are presented as mean ± SD, and different letters (a, b) indicate significant differences among groups (two-sided ANOVA test), P (a, b) < 0.05. **E, F** Inhibition of apoptosis by Z-VAD-fmk could not rescue oocyte loss induced by inhibiting FGFR1. The mouse ovaries were stained for DDX4 (green) and Hoechst (blue) after 6 days of culture. Scale bars: 50 μm **(E)**. Quantification of the oocytes per ovary after 6 days of culture. n=15. Mice ovaries were taken from at least 3 biologically independent experiments. Data are presented as mean ± SD, and different letters (a, b) indicate significant differences among groups (two-sided ANOVA test), P (a, b) < 0.05 **(F)**.


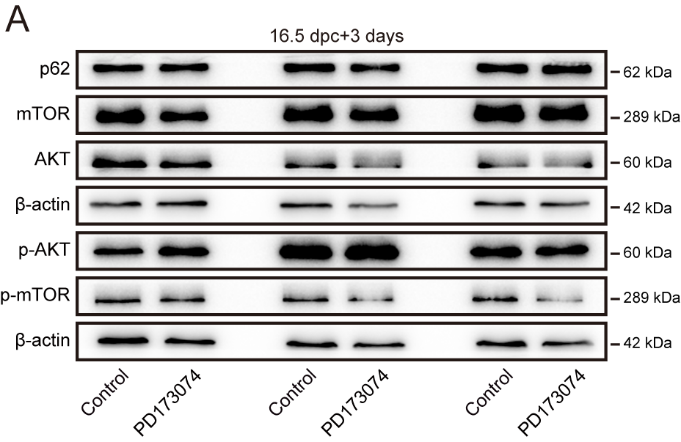


**Fig. S11 FGFR1 did not affect the activity of mTOR signaling pathway**

**A** Western blotting results showed that AKT/p-AKT, mTOR/p-mTOR and p62 were unchanged in PD173074-treated group compared with those in the respective controls.


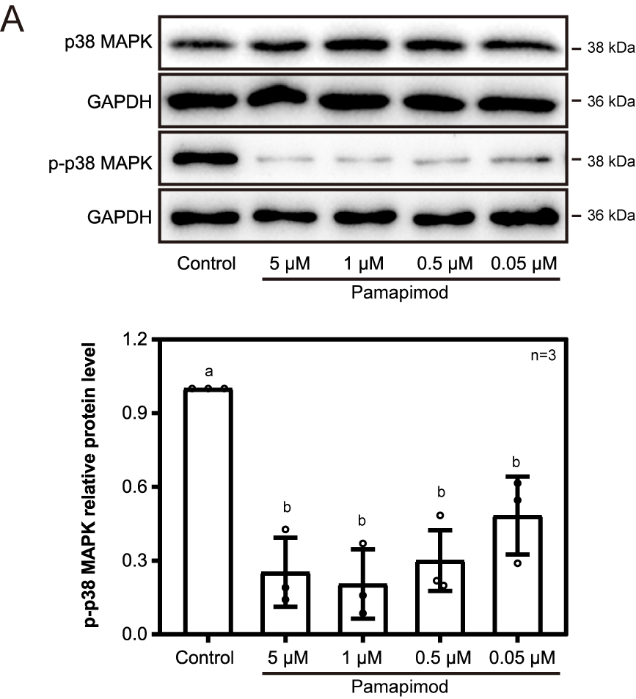


**Fig. S12 Inhibition efficiency analysis of Pamapimod.**

**A** Pamapimod significantly suppressed the activity of p38 MAPK in cultured ovaries. n=3 biologically independent experiments. Data are presented as mean ± SD, and different letters (a, b) indicate significant differences among groups (two-sided ANOVA test), P (a, b) < 0.05.


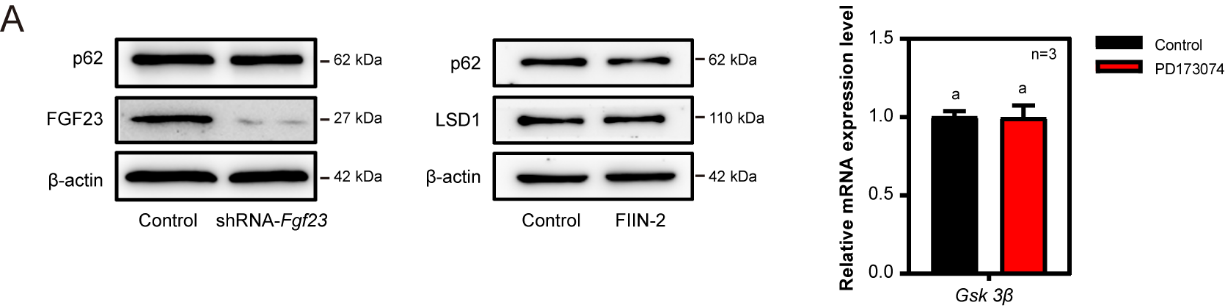


**Fig. S13 Changes in protein levels after *Fgf23* knockdown or FGFRs inhibition.**

**A** No significant change in p62 protein level after knockdown of *Fgf23* and no change in LSD or p62 protein level and no change in relative mRNA expression level of *Gsk 3β* after FGFRs inhibition. n=3 biologically independent experiments. Data are presented as mean ± SD (two-sided ANOVA test).

Table S1.

| Gene | Primer (5’ to 3’) |
| --- | --- |
| ShRNA-*Fgf23*-1-F | TCGAGCTATCACCTACAGATCCATATTCAAGAGATATGGATCTGTAGGTGATAGCTTTTTTGGTACC |
| ShRNA-*Fgf23*-1-R | GATCGGTACCAAAAAAGCTATCACCTACAGATCCATATCTCTTGAATATGGATCTGTAGGTGATAGC |
| *p38 Mapk*-F | CCGAACGATACCAGAACCTGTC |
| *p38 Mapk* -R | ACGCAACTCTCGGTAGGTCCTT |
| *Raf1*-F | CTTCAGGAACGAGGTGGCTGTT |
| *Raf1*-R | TGCTGCCTTCACACCACTGAGT |
| *Rasa1*-F | GCAAAACCCAGTATGGTCAGAGG |
| *Rasa1*-R | GCAAAACCCAGTATGGTCAGAGG |
| *Mapk3*-F | GGCTTTCTGACGGAGTATGTGG |
| *Mapk3*-R | GTTGGAGAGCATCTCAGCCAGA |
| *Mapk1*-F | TCAAGCCTTCCAACCTCCTGCT |
| *Mapk1*-R | AGCTCTGTACCAACGTGTGGCT |
| *β-actin*-F | GTGACGTTGACATCCGTAAAGA |
| *β-actin*-R | GCCGGACTCATCGTACTCC |
| *Tnf*-F | GGTGCCTATGTCTCAGCCTCTT |
| *Tnf*-R | GCCATAGAACTGATGAGAGGGAG |
| *NFκB*-F | GCTGCCAAAGAAGGACACGACA |
| *NFκB*-R | GGCAGGCTATTGCTCATCACAG |
| *Jnk1*-F | CGCCTTATGTGGTGACTCGCTA |
| *Jnk1*-R | TCCTGGAAAGAGGATTTTGTGGC |
| *Jnk2*-F | GTCAGTGGGTTGCATCATGGGA |
| *Jnk2*-R | ACTCTGCGGATGGTGTTCCTAG |
| *CJun*-F | CCTTGAAAGCTCAGAACTCGGAG |
| *CJun*-R | TGCTGCGTTAGCATGAGTTGGC |
| *Bax*-F | AGGATGCGTCCACCAAGAAGCT |
| *Bax*-R | TCCGTGTCCACGTCAGCAATCA |
| *Bcl2*-F | CCTGTGGATGACTGAGTACCTG |
| *Bcl2*-R | AGCCAGGAGAAATCAAACAGAGG |

Table S2.

| Antibody | Host | WB | IF/IHC | Catalog |
| --- | --- | --- | --- | --- |
| LC3B | Rabbit | 1:500 | 1:100 | Cell Signaling Technology, 2775 |
| p62 | Mouse | 1:500 | 1:100 | Abcam, ab56416 |
| mTOR | Rabbit | 1:500 | - | Cell Signaling Technology, 2983 |
| p-mTOR | Rabbit | 1:500 | - | Cell Signaling Technology, 5536 |
| Caspase-3 | Rabbit | 1:500 | - | Cell Signaling Technology, 9662 |
| Active caspase-3 | Rabbit | 1:500 | 1:100 | Beyotime Biotechnology, AC033 |
| DDX4 | Rabbit | 1:1000 | 1:300 | Abcam, ab13840 |
| DDX4 | Mouse | 1:1000 | 1:300 | Abcam, ab27591 |
| Foxl2 | Goat | 1:500 | 1:200 | Novus Biologicals, IMG-3228 |
| β-actin | Mouse | 1:1000 | - | CWbiotech, CW0098M |
| SYCP3 | Rabbit | - | 1:100 | Novus Biologicals, NB300-232 |
| γH2AX | Mouse | 1:1000 | 1:100 | Cell Signaling Technology, 80312 |
| FGF23 | Rabbit | 1:500 | 1:100 | Abmart, TD3596 |
| FGF23 | Rabbit | 1:500 | - | Invitrogen, **PA5-49926** |
| FGFR1 | Mouse | 1:500 | - | Invitrogen, **13-3100** |
| FGFR1 | Rabbit | 1:500 | 1:100 | Abmart, TA6156 |
| p-FGFR1 | Rabbit | 1:500 | - | Abmart, TA3157 |
| FGFR2 | Rabbit | 1:500 | - | Invitrogen, **PA5-96049** |
| FGFR3 | Rabbit | 1:500 | - | Invitrogen, **PA5-34574** |
| FGFR4 | Goat | 1:500 | - | Invitrogen, **PA5-47282** |
| P38 MAPK | Rabbit | 1:500 | - | Cell Signaling Technology, 8690 |
| p-P38 MAPK | Rabbit | 1:500 | - | Cell Signaling Technology, 4511 |
